# The MAGnetometers for Innovation and Capability (MAGIC) Technology Demonstration Payload

**DOI:** 10.1007/s11214-025-01191-5

**Published:** 2025-07-04

**Authors:** David M. Miles, Amanda Lasko, Scott Bounds, Matthew J. Blandin, Robert Broadfoot, Hansen Christian, Jeff Dolan, Richard Dvorsky, Matthew G. Finley, Allison M. Flores, Kenton Greene, Garret Hinson, Samuel Hisel, Connor Hogan, Jason Homann, Craig A. Kletzing, Katherine J. Morris, Chris Piker, Quentin Smith, Kevin L. Steele, Robert J. Strangeway, Antonio Washington

**Affiliations:** 1 Department of Physics and Astronomy, The University of Iowa, Iowa City, IA USA; 2 NASA Goddard Space Flight Center, Greenbelt, MD USA; 3 Department of Astronomy, University of Maryland, College Park, MD USA; 4 Space Sciences Laboratory, University of California, Berkeley, CA USA; 5 Department of Earth, Planetary and Space Sciences, University of California Los Angeles, Los Angeles, CA USA

**Keywords:** MAGIC, TRACERS, Magnetometer, Fluxgate, NASA, Heliophysics

## Abstract

The MAGnetometers for Innovation and Capability (MAGIC) instruments on the Tandem Reconnection And Cusp Electrodynamics Reconnaissance Satellites (TRACERS) Small Explorers (SMEX) mission are a technology demonstration (NASA NPR7120.8A). MAGIC will flight-demonstrate new low-noise fluxgate magnetometer cores, used to modulate the local in-situ magnetic field so that it can be sensed using the fluxgate technique, that were built from scratch at the University of Iowa Department of Physics and Astronomy. These fluxgate cores are intended to replace the dwindling supply of legacy Infinetics S1000 cores used historically in many spaceflight magnetometer applications. Each TRACERS spacecraft carries a technology demonstration magnetometer mounted on a common bracket with the TRACERS science magnetometer allowing direct in-flight validation of the new fluxgate technology. One TRACERS spacecraft carries a sensor that uses a backwards compatible 1” ring-core suitable for use in current magnetometer designs. The other TRACERS spacecraft carries a new sensor design called the Tesseract that uses race-track geometry fluxgate cores.

## Introduction

MAGnetometers for Innovation and Capability (MAGIC) is a fluxgate magnetometer Technology Demonstration (NASA NPR7120.8A) on the Tandem Reconnection And Cusp Electrodynamics Reconnaissance Satellites (TRACERS) Small Explorers (SMEX) mission (Miles et al. 2025a). TRACERS will study the spatial and temporal variation of dayside magnetic reconnection using transits through the Northern magnetospheric Cusp. The two TRACERS spacecraft will operate in a 590 km sun synchronous orbit in a ten to 120 second follow-the-leader configuration. Fluxgates have flown on a large percentage of all space science missions. However, reliance on legacy Infinetics S1000 fluxgate ring-cores (out of production since 1996) has generally constrained sensor designs. Many of the legacy ring-core supplies have been consumed forcing some providers to either use lower-grade cores or consider reclaiming cores from engineering models and flight-spares from previous missions. MAGIC flight demonstrates new, purpose-built fluxgate cores that do not rely on any legacy materials, processes, or hardware.

Section 1 summarizes the origin and intention of the MAGIC technology demonstration opportunity. Section 2 describes the as-built instruments and their major subsystems. Section 3 captures issues discovered during development and their mitigations. Section 4 describes the pre-flight calibration. Section 5 explains the accommodation on the TRACERS spacecraft and the instruments’ operation. Section 6 describes the planned science data products. Section 7 summarizes the magnetic noise removal techniques that are intended to be used in post-processing. Section 8 captures the benefits of this kind of technology demonstration flight. Finally, Sect. 9 concludes with the outcomes of the MAGIC project so far.

### New Instrument Design

Fluxgate magnetometers are a workhorse instrument for solar-terrestrial, Earth-observation, planetary, and operational space weather missions. However, the capacity to deliver high-quality space-flight magnetometers is constrained by the availability of high quality fluxgate cores. The University of Iowa has invested in new infrastructure that can implement a reverse-engineered process (Miles et al. 2019) for manufacturing new fluxgate cores that replace the legacy rings manufactured in the 1960s that have been used in many fluxgates to date. This manufacturing process was validated and optimized as part of the MAGIC project and the resulting hardware passed thermal vacuum and vibration qualification to establish a proven and renewable source of low-noise fluxgate cores for future instruments.

**Fig. 1:**
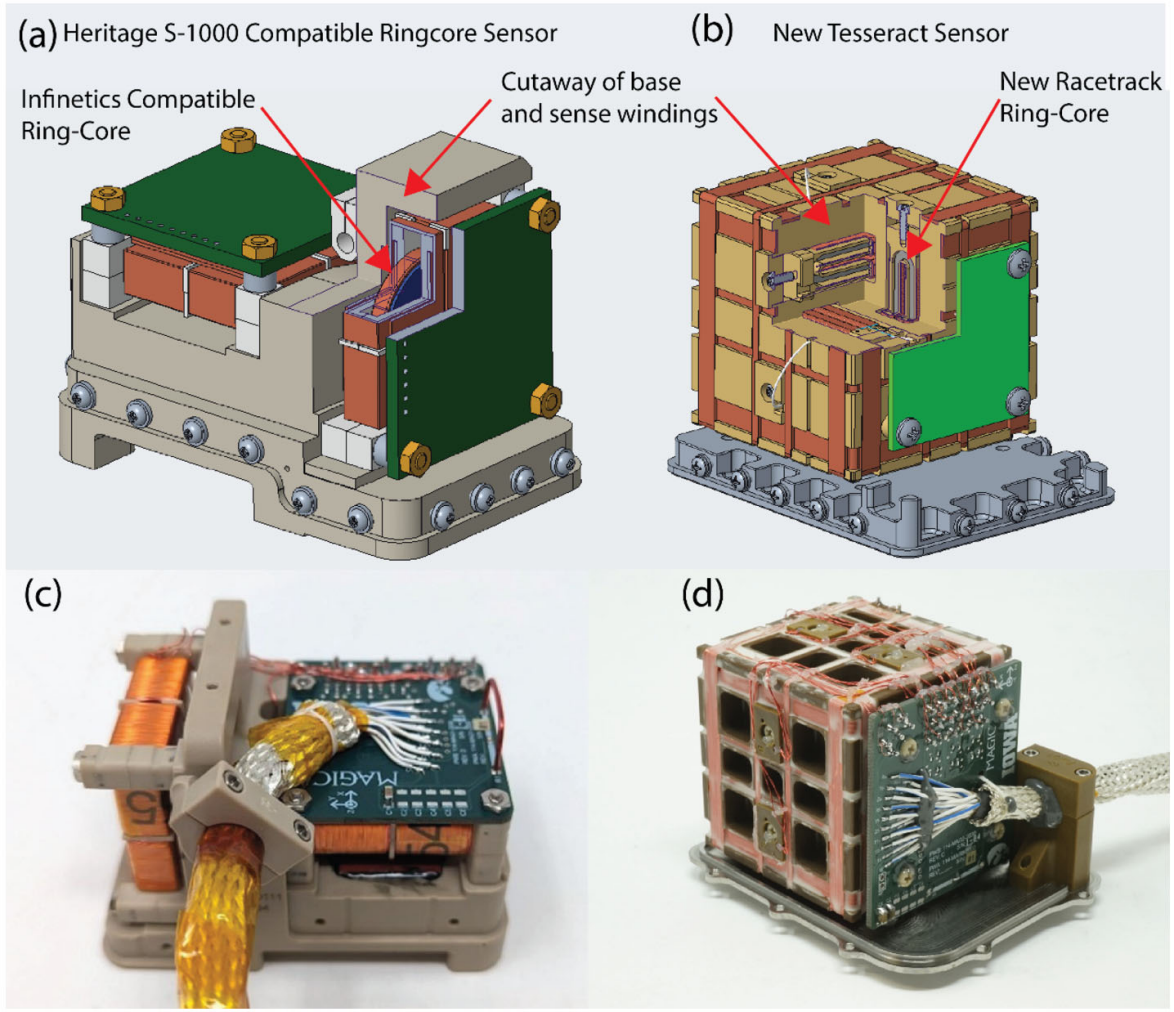
($a$,$c$) S1000 ring-core compatible fluxgate sensor. ($b$,$d$) Tesseract fluxgate sensor. Cutaways shows ferromagnetic cores and drive winding

The TRACERS mission comprises two spacecraft and, as a Technical Demonstration, MAGIC is not required for science closure on either. This provided an opportunity to demonstrate a lower TRL, but potentially higher stability and lower noise sensor on one spacecraft. All sensors are based on new low-noise fluxgate cores manufactured at University of Iowa (Miles et al. 2017, 2019; Narod and Miles 2024). MAGIC will demonstrate two sensor variants (Fig. 1) - one per spacecraft. The classic sensor (Fig. 1, a/c) is compatible with the classic ring-core geometry such as used by Mario Acuña at NASA Goddard (e.g., Acuña et al. 1978). The other spacecraft carries a new Tesseract sensor (Fig. 1, b/d) variant (Greene et al. 2022).

Both sensors have comparable mass and volume, use a common mounting interface, and share a common electronics design. Significant Phase B effort was spent optimizing the core manufacturing and calibration processes to ensure consistent performance for this and future missions. Both sensors underwent qualification testing to establish their readiness for spaceflight applications.

The MAGIC classic sensor design (Fig. 1, a/c) uses the same standard S-1000 geometry ring-cores as many historical spaceflight fluxgates and correspondingly has the same general dimensions and performance. This design had not previously been built up for a high-reliability spaceflight application at the University of Iowa. The design started from that flown successfully on the Investigation of Cusp Irregularities (ICI-5) sounding rocket in November 2019. Materials and EEE parts quality reviews were conducted to upgrade the design from using Commercial Off-The-Shelf (COTS) parts to those suitable for a high-reliability spaceflight application. Two engineering models were manufactured – the first from COTS parts and the second using flight-representative parts. Standard qualification testing (thermal vacuum, vibration, and electromagnetic interference) was performed to validate the instrument’s readiness for use in space.

### Flight Demonstration Summary

The MAGIC flight demonstration comprised the construction, qualification, and flight of magnetometer hosted payloads on the University of Iowa led TRACERS SMEX mission. MAGIC is flown in parallel with the originally proposed UCLA magnetometer (Strangeway et al, Current Issue) on a do-no-harm basis. TRACERS cannot rely on MAGIC to meet its science objectives, since MAGIC is a technology demonstration, however, MAGIC’s flight provides some resiliency in case of failure of the primary magnetometer, enables cross-comparison and validation, and provides an opportunity for gradient-driven signal processing to remove local magnetic noise.

The MAGIC flight payload consists of two fluxgate magnetometer instruments that measure the DC and low-frequency magnetic field. MAGIC-1 uses a sensor based on the traditional 1” ring-core geometry while MAGIC-2 uses the novel Tesseract sensor design (Greene et al. 2022, 2024). MAGIC adhered to NASA’s NPR 7120.8A standard for technology demonstration, except where it directly interfaces with the host mission. There it adhered to the standards of NPR 7120.5F to ensure it did not have a negative impact on the mission. MAGIC followed a do-no-harm approach appropriate for a Technical Demonstration hosted-payload.

**Fig. 2:**
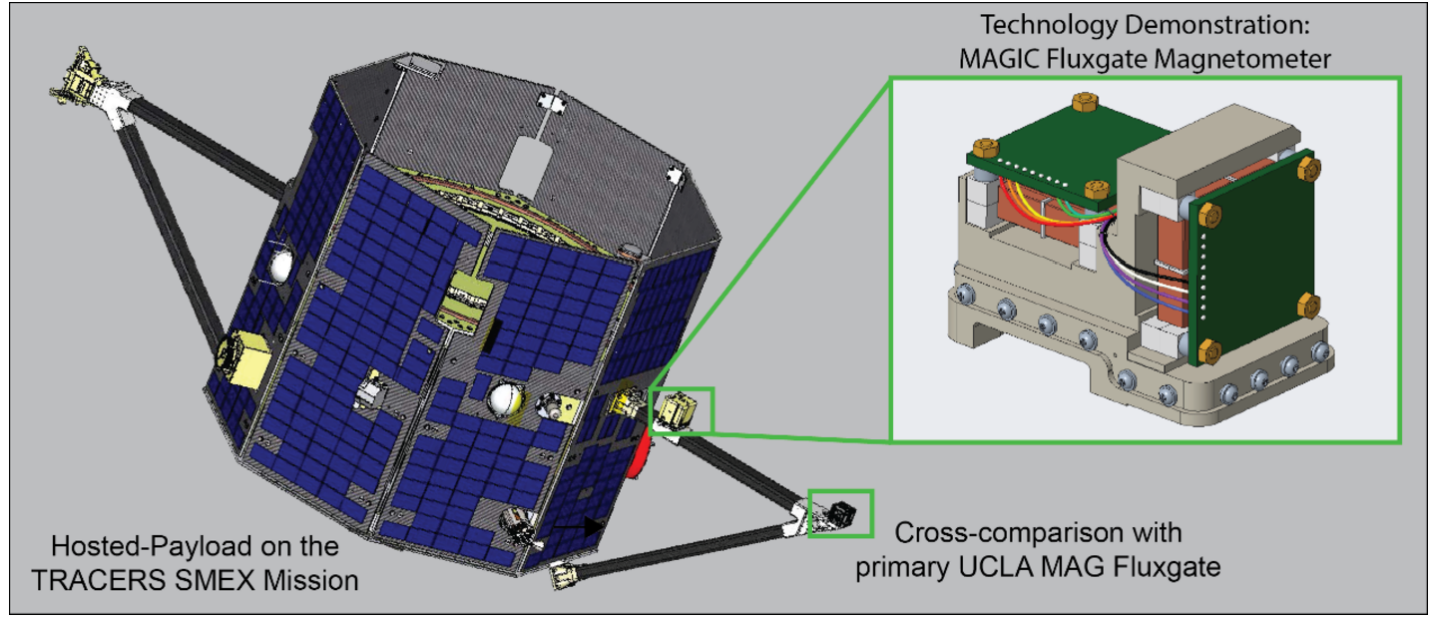
The MAGIC sensor is accommodated on the same magnetometer bracket as the primary MAG fluxgate in the TRACERS science instrument payload. Spacecraft image credit Millenium Space Systems

MAGIC is mounted 20 cm along the 70 cm primary MAG magnetometer bracket (Fig. 2), allowing differential measurements of the magnetic noise of the spacecraft. The MAGIC electronics are accommodated as a small, independent electronics box mounted to the side of the TRACERS Main Electronics Box (MEB). MAGIC interfaces electrically through the MEB developed at University of Iowa, allowing in-house interface testing, minimizing the additional demands imposed on the spacecraft, and ensuring MAGIC can be powered off in the event of on-orbit issues. The Magnetic Search Coil (MSC) magnetometer, mounted on the opposite bracket, is heavier than the existing MAG fluxgate so the MAGIC sensor was accommodated without increasing the total bracket assembly mass, as MAGIC replaced existing spin-ballast.

Ferromagnetic material and magnetic feedback in both fluxgate sensors will modestly affect each other’s measurements. This was characterized before launch for post-processing correction (Miles et al. 2025b) and the two instruments are synchronized, using failure tolerant logic, to prevent the fluxgate drive signals from beating against each other. Vector measurements at 128 sps from both instruments are telemetered during the region of interest, allowing differential measurement signal processing to mitigate the stray spacecraft magnetic field in post-processing without the risk of lossy on-orbit data reduction. MAGIC operates from a single switched 28 V service controlled by the TRACERS Central Data Processing Unit (CDPU), allowing it to be powered off if something goes wrong. When powered-off, MAGIC is completely removed from the standard operation of the spacecraft except for a small (<1 nT) and predictable stray field from the ferromagnetic cores. This was quantified during pre-flight testing and is removed in post processing.

### Requirements

MAGIC matched its measurement requirements to those defined by the host TRACERS mission Fluxgate Magnetometer (MAG) science payload. MAG contributes to all three of the TRACERS mission science objectives (Miles et al. 2025a). Tracing the science objectives to the magnetic observables, gives the driving instrument requirements shown in Table 1. Although MAGIC does not formally contribute to TRACERS science closure, MAGIC data could serve the same three scientific purposes as the primary MAG payload – help measure the magnetic field convection velocity, establish the background field direction for particle pitch-angle distribution calculation, and measure the magnetic component of low-frequency plasma waves. Estimating the convection velocity requires measurements of the field with a cadence of at least 10 sps with an accuracy of 100 nT. This accuracy is more than sufficient to meet the angular resolution of the particle instruments. The performance listed in Table 1 of < 10 nT is limited by the available test facilities for measuring absolute accuracy. Actual instrumental accuracy will be determined on-orbit using in-situ calibration compared to the CHAOS magnetic field model (Finlay et al. 2020).

**Table 1:** Traceability to the TRACERS Science Measurement Requirements

TRACERS measurement requirement		Instrument requirement	MAGIC performance
Physical parameter	Observable		
Cusp magnetic field convection Velocities (S01)	DC Vector Magnetic Field	Temporal Resolution: 0.1 s	Temporal Resolution: 0.0078 s
		Accuracy: 100 nT	Accuracy: 10 nT
Convection Velocities (S02)	DC Vector Magnetic Field	Temporal Resolution: 0.1 s	Temporal Resolution: 0.0078 s
		Accuracy: 100 nT	Accuracy: <10 nT
Alfvén And Other Wave Signatures (S03)	Wave AC Magnetic Field (MAG Portion)	Frequency Response: DC - 10 Hz	Frequency Response: DC - 50 Hz
		Resolution (MAG): 1 nT	Resolution: <0.5 nT

Resolving Alfvén and other wave signatures requires a sensitivity of at least 1 nT up to 10 Hz. The MAGIC design provides sensitivity that is essentially invariant with frequency (cf., Primdahl et al. 1994) resulting in a noise floor that is flat at higher frequencies, set by the resolution of the forward analog loop and the digitizer, and dominated by core noise below ∼1 Hz.

The MSC search coil is more sensitive at higher frequencies and will be used to observe higher frequency wave modes. MAGIC provides vector magnetic field measurements at 128 samples per second with noise levels of less than 10 pT/$\sqrt{}$Hz at 1 Hz. MAGIC is designed to maintain resolution of less than 0.5 nT at all points in the orbit in full Earth-field of ±65,000 nT.

Operating on the TRACERS spacecraft imposes several additional requirements:

- Operate in full Earth field at the TRACERS orbit altitude of 590 km.
- Sufficiently high bandwidth to track the spacecraft spin rate of 2 - 10 rpm.
- Sensor mass (< ∼500 g) suitable for accommodation on the TRACERS magnetometer bracket.

## MAGIC Instrument Description

### Overview

**Fig. 3:**
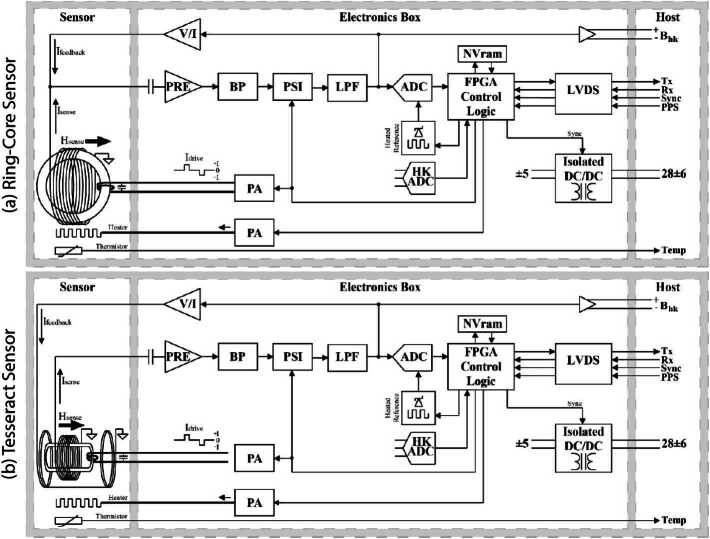
Simplified single axis MAGIC block diagram illustrating ($a$) the classic ring-core design and ($b$) the new Tesseract sensor design

The MAGIC instruments flown on each spacecraft differ to demonstrate different alternative technology solutions. The first MAGIC sensor uses a high-heritage ring-core geometry design (Fig. 1, a/c) while the other uses the new ‘Tesseract’ (Greene et al. 2022, 2024) sensor design (Fig. 1, b/d). The two sensors implement magnetic feedback differently. The classic ring-core sensor uses solenoidal windings to both sense the modulated magnetic field at AC and provide magnetic feedback at DC (Fig. 3, a). Conversely, the Tesseract sensor uses solenoidal windings on each core to sense the modulated field but provides magnetic feedback through separate Merritt coil windings (Fig. 3, b).

### Design Considerations

The MAGIC payload considered several constraints during its design and construction.

The MAGIC sensor is subject to electromagnetic interference at both baseband (0 to 64 Hz) and at the second harmonic of the nominal drive frequency 16,384 Hz ± 64 Hz. Transient electrical signals due to phenomena including the illumination of solar panels, battery charge/discharge currents, or subsystems powering on or off can generate detectable magnetic noise. The MAGIC team participated in the TRACERS magnetic cleanliness control board to guide mission-level magnetic cleanliness processes (screening for remnant magnetic fields, ferromagnetic materials, current loops, back-wiring solar panels, etc.). MAGIC provided additional staff to help implement the electromagnetic cleanliness control measures (Miles et al. 2025b). This included DC screening of piece parts and subsystems, a magnetic swing AC screening of instruments and spacecraft subsystems, and a magnetic swing test of each completed spacecraft to bound their dipole moments (Miles et al. 2025b).

The MAGIC sensors, designed to operate on deployable booms or fixed non-magnetic brackets, are robust to radiation effects and require minimal EEE parts (platinum temperature sensor and capacitors). The paired electronics packages are designed such that they can be manufactured with a minimum radiation Total Integrated Dose (TID) tolerance of >50 krad (exceeding the expected 4.4 krad TRACERS mission TID) and a single event latch up LET of >10 MeV/cm^2^/mg, with two notable exceptions. The ProAsic3 A3PE3000-PQG208 FPGA and the RT2378-20 ADC were not rated for >50 krad of radiation. However, the A3PE has a direct upgrade in the RT3PE3000 which is rated for 25 krad, but the A3PE was deemed acceptable for the mission due to the low overall expected on orbit TID. The RT2378-20 is rated for 10 krad, which is still over the expected mission TID, but not the desired minimum.

### Fluxgate Core Design

Fluxgate magnetometers (Primdahl 1979; Snare 1998) sense the magnetic field by the electromagnetic force $V_{i}$ induced in a coil of $N$ turns, area $\boldsymbol{A}$, field $\boldsymbol{H}$ by the changing magnetic flux created by periodically saturating a ferromagnetic core of relative permeability, $\mu _{r}$. This is reasonably approximated by $V_{i} \approx \left ( N \mu _{0} \boldsymbol{A} \cdot \boldsymbol{H} \right ) \frac{d \mu _{r}}{dt}$. The noise of the magnetic field measurement is often dominated by the intrinsic magnetic noise of the ferromagnetic core as it cycles through magnetic saturation. Much of the primary research into manufacturing low-noise fluxgate cores was done for military sensing purposes and subsequently lost to the public literature. Consequently, many spaceflight fluxgate magnetometers use legacy magnetic materials manufactured by the US Naval Surface Weapons Center (NSWC) White Oak and Infinetics Inc. (Scarzello et al. 2001). The providence of these materials is poorly documented as are the techniques by which they were built. In contrast, MAGIC infuses the results of recent work to understand the origin of fluxgate noise and to develop and publish a repeatable process for manufacturing high-quality fluxgate cores (Miles et al. 2019, 2022; Narod 2014; Narod and Miles 2024).

The MAGIC Ring-Core sensor is based on a reverse-engineered 6% Molybdenum permalloy similar to that used in the legacy cores (Miles et al. 2019) and uses a spiral-wound 1” ring-core geometry compatible with the Infinetics S1000 ring-core. In contrast, the MAGIC Tesseract sensor is based on a new Copper Permalloy (Miles et al. 2022; Narod and Miles 2024) and uses racetrack geometry build up from stacked continuous washers of permalloy foil.

**Fig. 4:**
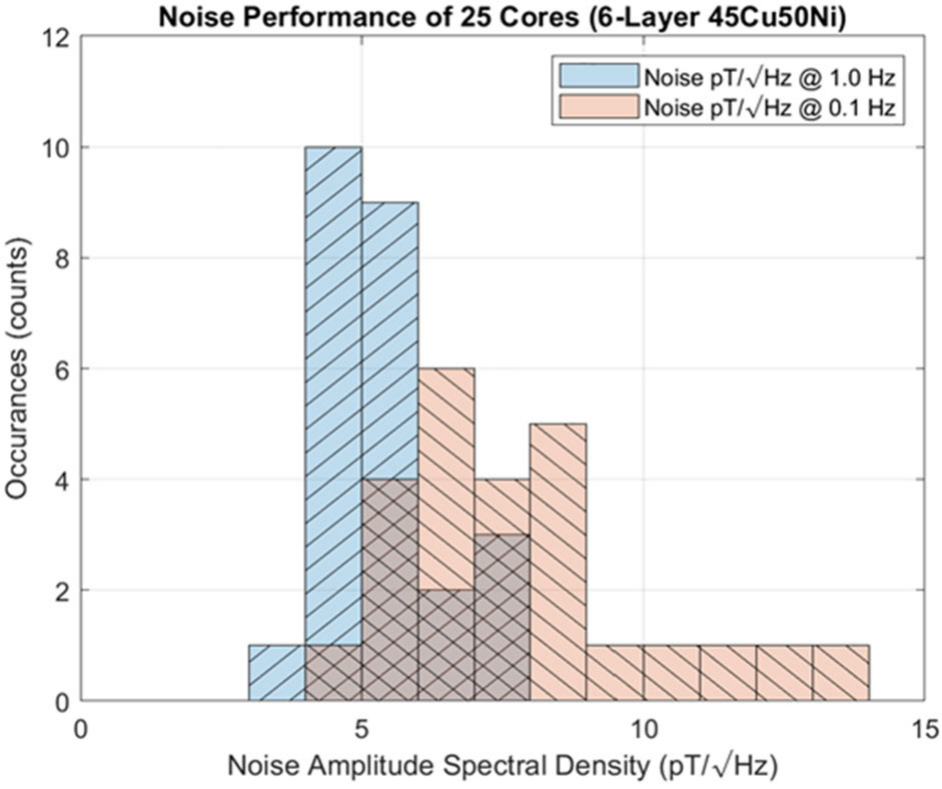
Histogram of noise for twenty-five 6-layer fluxgate cores manufactured from 45% copper, 50% nickel, iron to fill. Noise is measured as power-spectral-density (PSD) at both 1.0 and 0.1 Hz. Cross-hatched columns indicate statistics for both test frequencies. Reproduced from Narod and Miles (2024)

Twenty-five 6-layer racetrack cores were manufactured to demonstrate the reproducibility of the new design and manufacturing process. Noise Power Spectral Density (PSD) results for these cores are presented in Fig. 4 demonstrating noise of 4-5 pT/$\sqrt{}$Hz at 1.0 Hz and 6-7 pT/$\sqrt{}$Hz at 0.1 Hz.

### Sensor Design

The primary difference between the two MAGIC sensors is the design of the fluxgate core and how the drive winding, sense and/or feedback windings are therefore applied.

#### Ring-Core Sensor Design

**Fig. 5:**
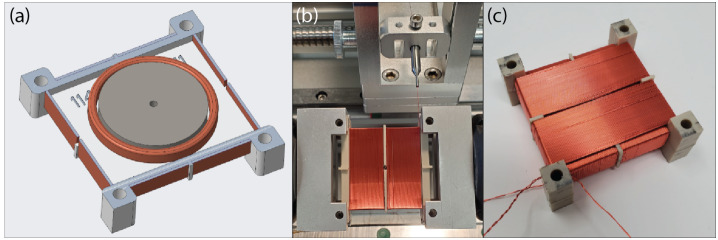
($a$) Render showing a new S1000 compatible ring-core centered within a bobbin that will support the sense-windings. ($b$) The internal sense winding is applied as two segments of rectangular solenoids on either side of a hole used to align the ring-core. ($c$) Finished ring-core assembly with outer sense-winding of two segments of rectangular solenoids applied orthogonal to the inside winding

The ring-core is driven through magnetic saturation using a continuous toroidal drive winding (Fig. 5, a). The wound core is centered in a rectangular bobbin that supports two nested solenoidal windings (Fig. 5, b, c) that both sense the two components of the modulated magnetic field and apply magnetic feedback.

**Fig. 6:**
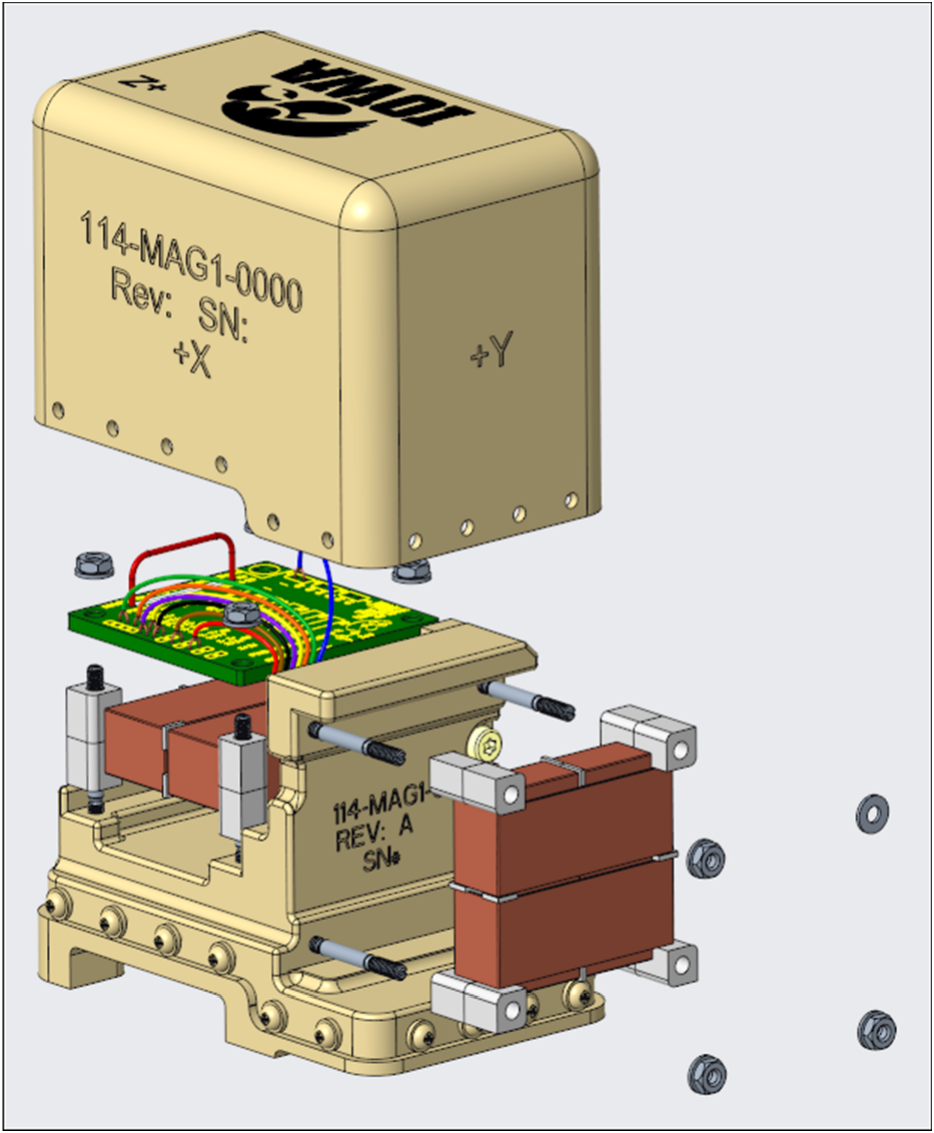
Exploded render of the ring-core sensor showing the base, two double-wound ring-core assemblies mounted orthogonally, printed wiring board for harness and magnet wire termination, and a protective cover

Each of these double-wound bobbin assemblies measures two orthogonal projections of the local magnetic field. A second double-wound bobbin assembly mounted to a common base (Fig. 6) completes the vector measurement – one axis being the series connector of the two aligned sense windings on the two bobbin assemblies. A small, printed wiring assembly terminates the various windings, connects the cable harness, hosts the capacitor bank which tunes the resonant drive circuit, and provides a non-magnetic platinum temperature sensor. A non-magnetic plastic housing closes out the sensor assembly, protects the delicate windings during integration, and provides some protection from ferromagnetic debris.

#### Tesseract Sensor Design

**Fig. 7:**
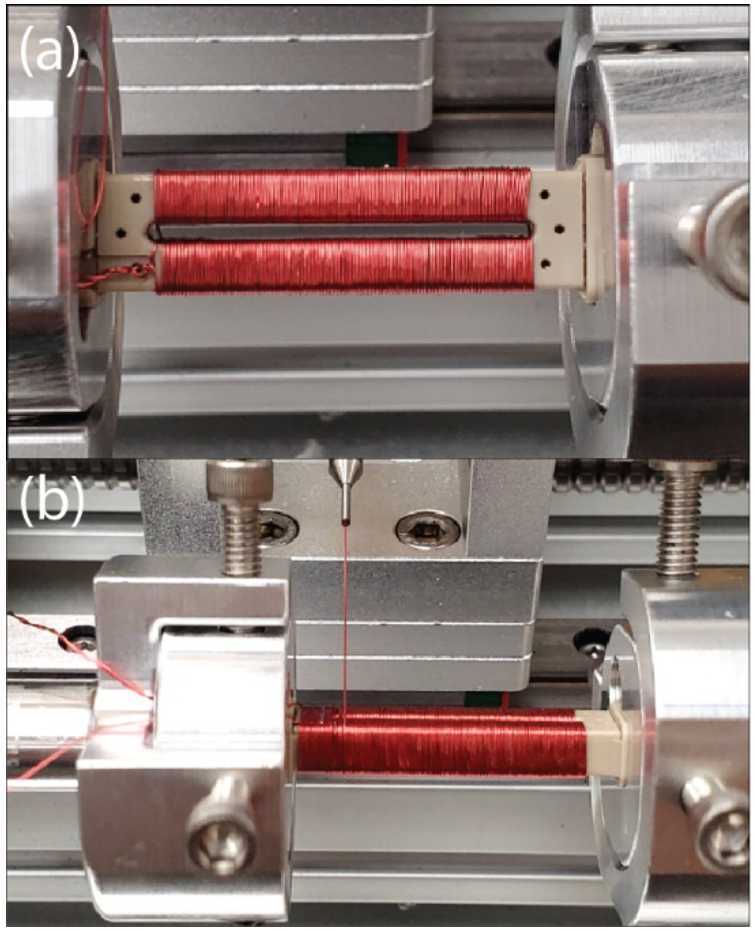
($a$) Pseudo-toroidal drive-winding applied to a race-track fluxgate core. ($b$) Solenoidal pickup winding being applied on top of the drive-winding

The Tesseract sensor was designed with two primary intents – to be compatible with the new racetrack fluxgate cores and to mitigate several known sources of fluxgate instability (orthogonality variation with temperature, residual field in the sensor, mechanical core changes, etc.). Tesseract leverages recent advances in low-noise custom fluxgate cores (Miles et al. 2019, 2022) and temperature compensation (Miles et al. 2017) to create a compact, rigid, symmetric, and thermally compensated sensor that provides high magnetic stability. The racetrack cores are driven through magnetic saturation using pseudo-toroidal windings that are placed by hand on each core (Fig. 7, a). The modulated magnetic field is sensed by a solenoidal winding (Fig. 7, b) applied on top of the drive windings using a CNC winder.

**Fig. 8:**
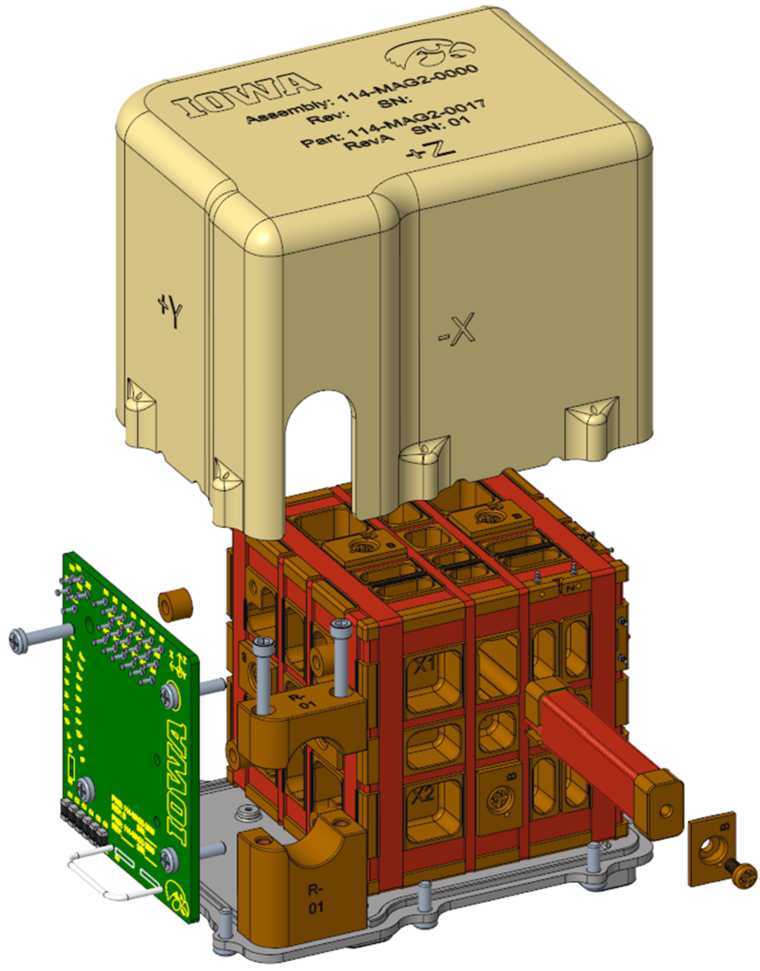
Exploded render of the Tesseract sensor showing one of the six racetrack cores, cubic base supporting three orthogonal Merritt coil feedback windings, a printed wiring assembly to terminate the magnet wire and harnessing, harness strain relief, titanium base with mounting features, and the protective lid

The Tesseract feedback windings are each constructed in a Merritt coil of four windings per axis (Fig. 8). The three orthogonal feedback windings allow the field to be nulled in all components (Primdahl and Jensen 1982) regardless of the ambient magnetic field. This is expected to improve linearity and stability as previous sensors zeroed in two directions (Wallis et al. 2015) perform better than those zeroed in only a single component (Acuña et al. 1978; Narod and Bennest 1990). The Merritt coil creates a larger homogeneous region and accommodates more cores (6 vs 3) than sensors using Helmholtz arrangements of two coils per axis (e.g., Forslund et al. 2007).

The bobbins and the sensor base are manufactured from a common material (glass filled Torlon) reducing the impact of mismatched coefficients of linear thermal expansions compared to the Inconel bobbin used in the S1000 ring-core. The three orthogonal feedback windings are formed symmetrically on a common structure. This reduces the tendency of the sensor to skew with temperature, preserving the orthogonality, and provides a linear thermal gain sensitivity that can be compensated in the electronics in post processing.

The Tesseract sensor assembly is completed by a printed wiring assembly to terminate the enameled wire from the various windings, strain relief for the sensor harness, a titanium plate providing the mounting interface, and a shell lid for protection.

### Electronics Design

**Fig. 9:**
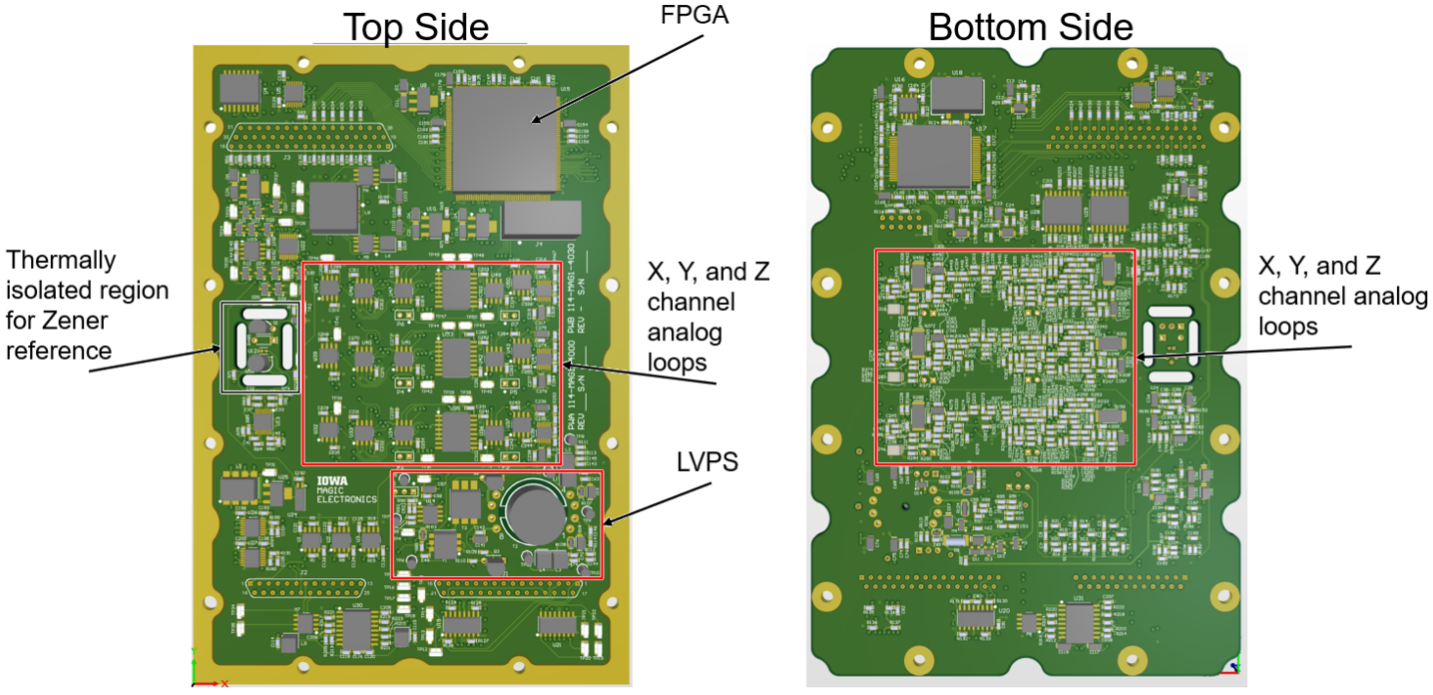
MAGIC printed wiring board render showing major instrument subsystems

The MAGIC electronics are implemented in a single high-density printed wiring assembly shown in Fig. 9. Control logic is provided by a configurable FPGA. The ferromagnetic core is periodically driven into magnetic saturation and the resulting signal is demodulated at the second harmonic. Global negative magnetic feedback, achieved using precision temperature compensated current source, linearizes the instrument response, and extends its magnetic range – the final measurement being primarily the amount of current required to null the magnetic field in each component. A temperature compensated precision Zener reference helps ensure absolute instrumental stability. A non-magnetic heater incorporated into the sensor is available for use on-orbit but is not expected to be required given thermal modeling.

#### Core Drive Subsystem

**Fig. 10:**
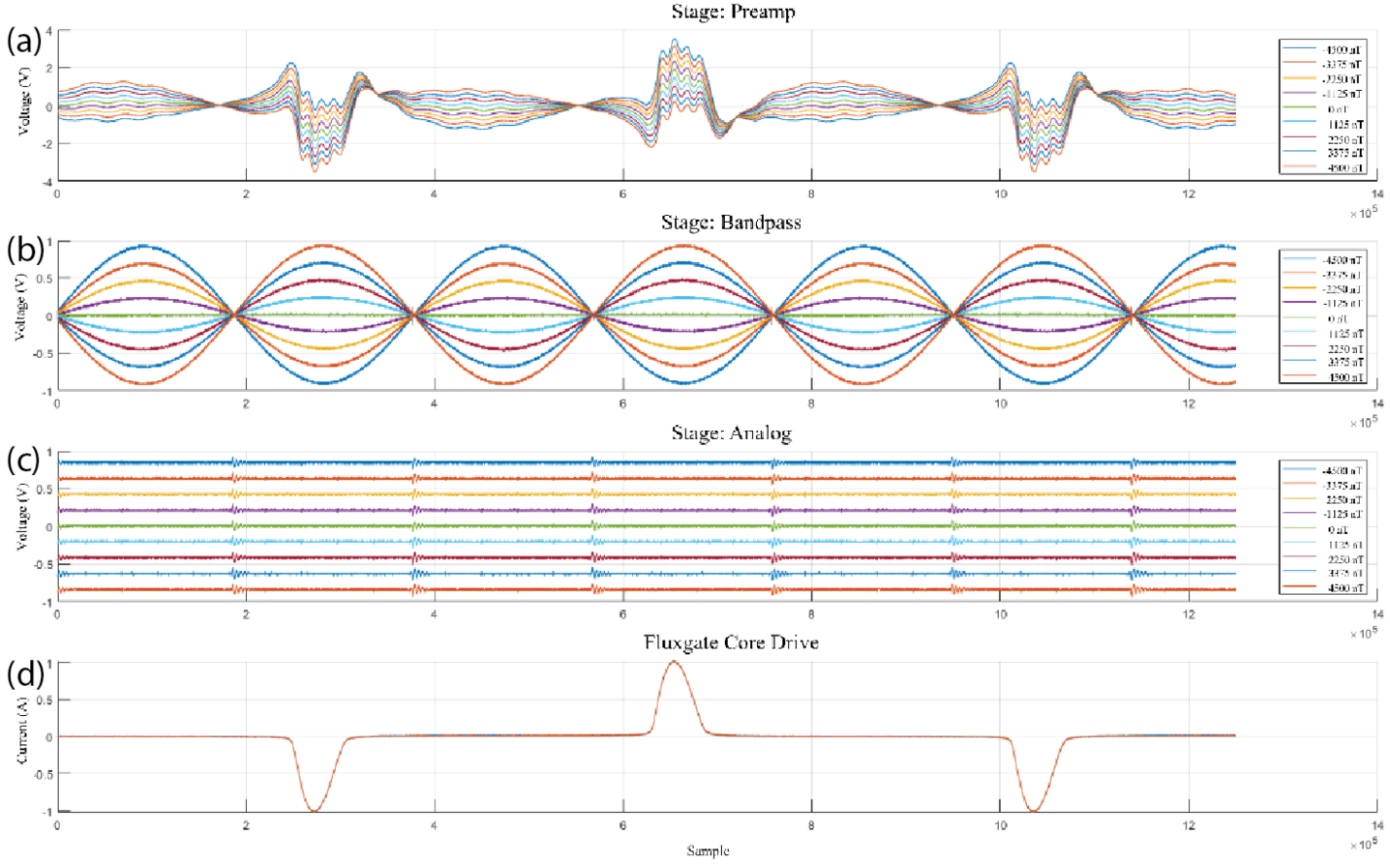
($a$) Alternating polarity current waveform used to drive the fluxgate cores through magnetic saturation. ($b$) Preamp output showing envelope of applied magnetic fields. ($c$) Filter chain out showing recovered 2F carrier. ($d$) Low pass filtered quasi-DC voltage proportional to applied magnetic field on that sensor’s axis

The sensor’s core drive windings are controlled through a MOSFET H-bridge on the electronics board controlled by four signals coming from the FPGA. These drive control signals are designed such that diagonal pairs of MOSFET gate “ON” signals, a serial common mode choke, tuning shunt capacitors, and the serially connected fluxgate cores to create a resonant circuit that generates alternate polarity current pulses to momentarily magnetically saturate the cores. Alternating polarity positive and negative pulses prevent a remanent magnetisation of the core through repeated exposure to one direction of magnetic stimulus. The drive is set at 8192 Hz (1F) with current pulses at twice that frequency (2F). The drive circuit was tuned such that the saturating current pulse (Fig. 10, a) is of sufficient amplitude to drive the cores into deep magnetic saturation, but of short duration to minimise the average power draw of the sensor.

#### Analog Loop and Demodulation

The forward analog loop of the electronics consists of a chain of amplifiers, filters, and switches to take the current output of the sense windings and convert them to a DC voltage that is proportional to the external magnetic field. That voltage is also converted into a current with the opposite polarity and driven back into the sense winding of the Ring-Core sensor (or the feedback winding of the Tesseract sensor) to apply global negative magnetic feedback.

The forward analog path converts a current from the sense winding to a voltage through a trans-impedance pre-amplifier. This pre-amplifier includes a parallel resistor-capacitor-inductor (RCL) feedback network that acts as a weak bandpass filter and is the only part of the chain that adds gain to the system. A low-noise RH1028 op-amp is used as this stage dominates the noise of the electronics. The remaining op-amps are low quiescent current ADA4084s or high output current ADA4610s.

The core drive signal couples into the sensor output – primarily at 8192 Hz (1F) and 24,476 Hz (3F) odd harmonics and needs to be removed before the signal is demodulated to baseband. A strong bandpass around 16,384 Hz (2F) would be ideal, but a sufficiently low noise design could not be found that also met power and temperature stability requirements. Instead, a three-stage filter consisting of two bandstop filters was designed to eliminate the two strongest drive harmonics, 1F and 3F, followed by a low pass filter with a -3 dB frequency of 18 kHz, to eliminate the subsequent, lower amplitude harmonics. Together, this filter recovers the 16,384 Hz (2F) carrier with an amplitude and polarity that is modulated by the magnetic field on that sensor axis.

The signal is demodulated by an analog switch whose input is synchronized in phase to the zero crossings of the filtered sense signal – splitting it into two phases. One phase is inverted and added back, effectively creating a full wave rectifier that recovers both the amplitude and the polarity of the input 1F carrier. A low pass filter at 50 Hz creates a quasi-DC voltage that is proportional to the magnetic field in that component of the sensor. This voltage is sampled by an ADC, decimated to the final data product rate, and sent to the TRACER Central Data Processing Unit (CDPU) as the MAGIC science data product. Figure 10 shows the various stages of the MAGIC electronics illustrating the drive (a), the voltage output of the preamp (b), the recovered 2F carrier (c), and the quasi-DC output proportional the magnetic field on that component.

The quasi-DC voltage that is sampled by the ADC is also converted back into a current by a trans-conductance amplifier and sent back into the sense winding of a Ring-Core sensor (or into the feedback windings of a Tesseract sensor). The trans-conductance amplifier is tuned to control the percentage of magnetic nulling in the sensor, which balances the range, linearity, temperature compensation, and stability of the system.

#### Sensor Heater

**Fig. 11:**
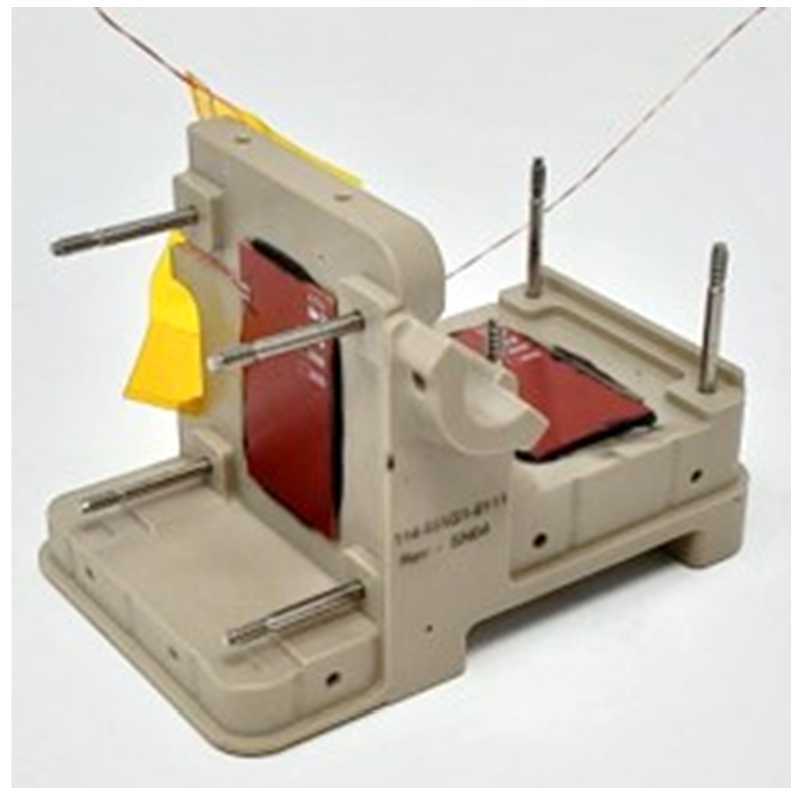
Non-magnetic purpose-built sensor heater boards thermally bonded to the ring-core sensor base prior to assembly

MAGIC includes a technology demonstration of a non-magnetic sensor heater comprising a purpose-built circuit board with no components but a single meandering trace spanning six layers, through which a current can be applied to generate up to ∼0.5 W of heat with a self-canceled magnetic field. The board is thermally bonded to the sensor base (Fig. 11) and terminated on the sensor board. The control circuit on the electronics consists of an H-bridge copied from the drive circuit (omitting the common mode choke) driven as a pulse-wide modulated signal with a repetition rate far above sensitive frequency range of MAGIC. A Proportional-Integral-Differential (PID) control loop in the FPGA can then heat the sensor to a programmable set-point. The sensor heater will be tested on-orbit after the primary science objectives have been achieved during the back-orbit period when TRACERS is not capturing science data in the magnetopsheric cusp.

**Fig. 12:**
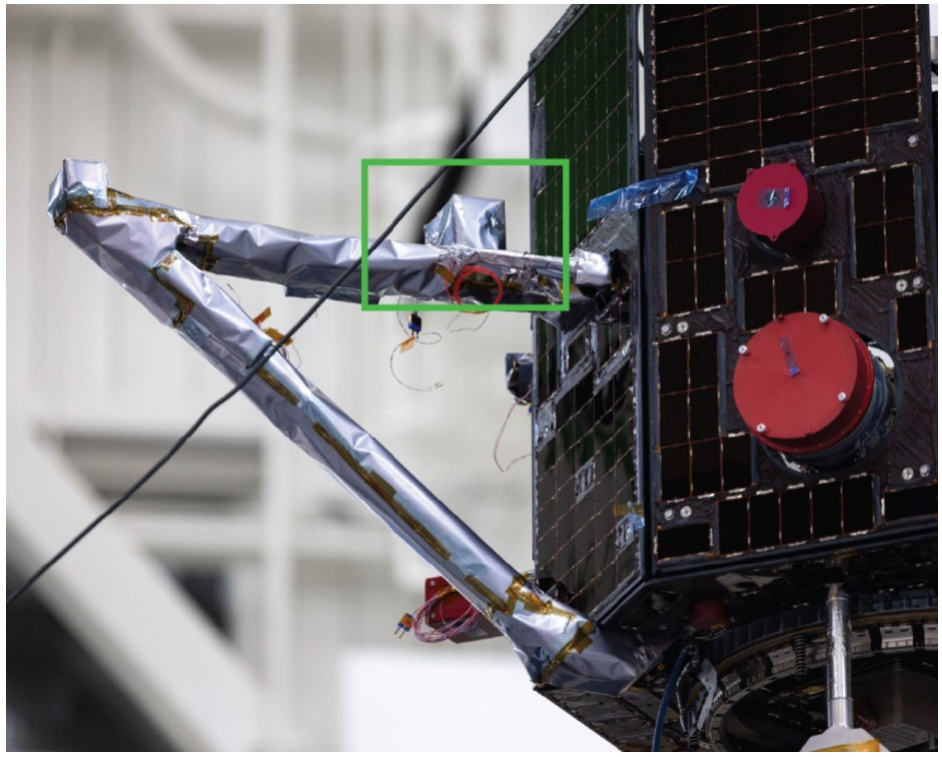
Each MAGIC sensor is encapsulated in Multi-Layer Insulation (MLI) blankets on the fixed magnetometer brackets

The thermal environment expected on-orbit is relatively benign. Each MAGIC sensor is mounted to a bracket on the carbon fiber rigid bracket, which is subsequently mounted to an interface plate on the spacecraft out bulkhead. The magnetometer brackets are encapsulated in purpose built Multi-Layer Insulation (MLI) blankets (Fig. 12). Combined with the modest power dissipated within the sensor, the expected on-orbit temperature range is -17 to +27 °C. Both sensors were thermally qualified from -32 to +54 °C during the spacecraft level thermal vacuum test campaign.

#### Zener Heater

**Fig. 13:**
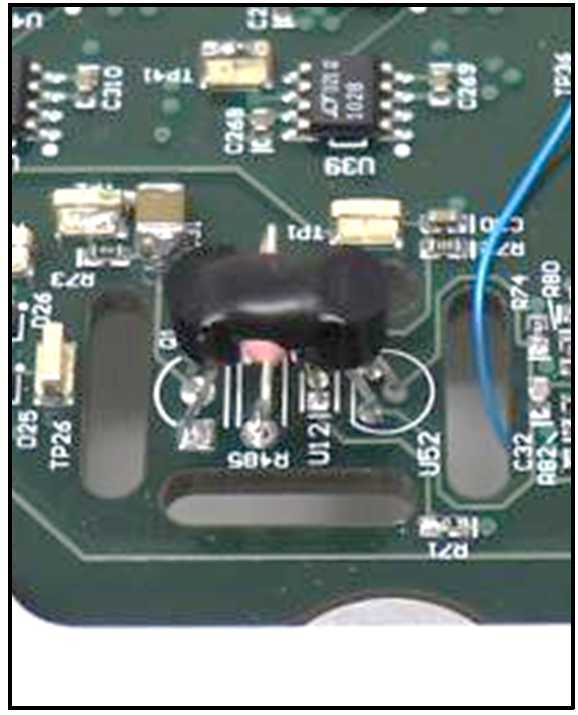
Implementation of Zener heater thermal isolation

The accuracy of the magnetic field measurements cannot exceed that of the RH129 precision reference, with a Zener voltage of 6.9 V, that is divided and buffered to ±3.45 V as the voltage limits of the analog sensor voltage range. The voltage output varies slightly with temperature so some commercial versions have a built-in thermally stabilized heater package that maintain a constant temperature. The radiation tolerant equivalent does not have this capability so MAGIC implemented a custom control loop to provide this functionality. The four components of the thermally stabilized reference (the RH129 Zener reference, a 10 $\Omega $ resistor for heating, a 2N2222 BJT to control the heater output, and an AD590 transducer to measure the temperature of the circuit) are raised above the board in a thermally isolated zone (Fig. 13) and bonded together using a thermally conductive epoxy.

A PID controlled pulse-width-modulation (PWM) duty cycle input to the BJT controlling the resistor’s heating is used with feedback from the temperature sensor to maintain the reference at a consistent programmable temperature above that predicted for the electronics on-orbit. The heater can apply up to ∼1.5 W of heat, but in practice far less power is required to stabilize the Zener temperature when the electronics are in vacuum due to the thermal isolation. The loop area of the heater circuit is sufficiently small that it generate no measurable stray magnetic field at the sensor. In practice, the PWM controller can maintain the Zener temperature within 1 °C, which makes the Zener’s intrinsic ∼10 ppm/°C temperature coefficient negligible compared to other instrumental effects.

### Clock Synchronization

MAGIC, as a do-no-harm payload, was required to fully mitigate its impact on the host TRACERS mission. The most challenging interaction to manage was the magnetic crosstalk between the MAGIC and MAG fluxgate sensors which are mounted ∼50 cm apart on a fixed rigid bracket (Miles et al. 2025b). The instruments in the TRACERS science payload were designed to run asynchronously. This poses a challenge for the two nearby fluxgate magnetometers as each sensor emits a small, but non-zero, time-varying stray magnetic field as it periodically drives it cores through magnetic saturation. This results in a beat-frequency in the magnetic data as the drive circuit phase of one instrument drifts past the sampling phase of the other instrument.

**Fig. 14:**
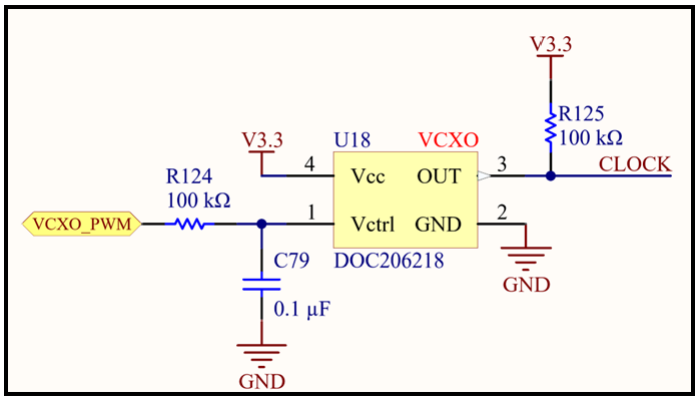
MAGIC uses a Proportional-Integral-Differential (PID) control loop and pulse width modulation (PWM) to steer its local oscillator to match the frequency and phase of the MAG fluxgate magnetometer and mitigate the effects of the magnetic interference generated by its core-drive

MAGIC mitigates this effect by synchronizing its operation to that of MAG. The MAG instrument transmits a Low-Voltage Differential Signaling (LVDS) signal that is phase locked to the 1F control of its core drive circuit. MAGIC generates its base clock using a voltage-controlled crystal oscillator (VCXO) and implements an FPGA driven PID control loop (Fig. 14) that steers its oscillator until it its local 1F drive signal matches MAG’s 1F in both frequency and phase. This was shown in laboratory testing to fully mitigate the drive signal beat frequency interference, even when the sensors were artificially close together (Miles et al. 2025b).

### FPGA Control Logic

**Fig. 15:**
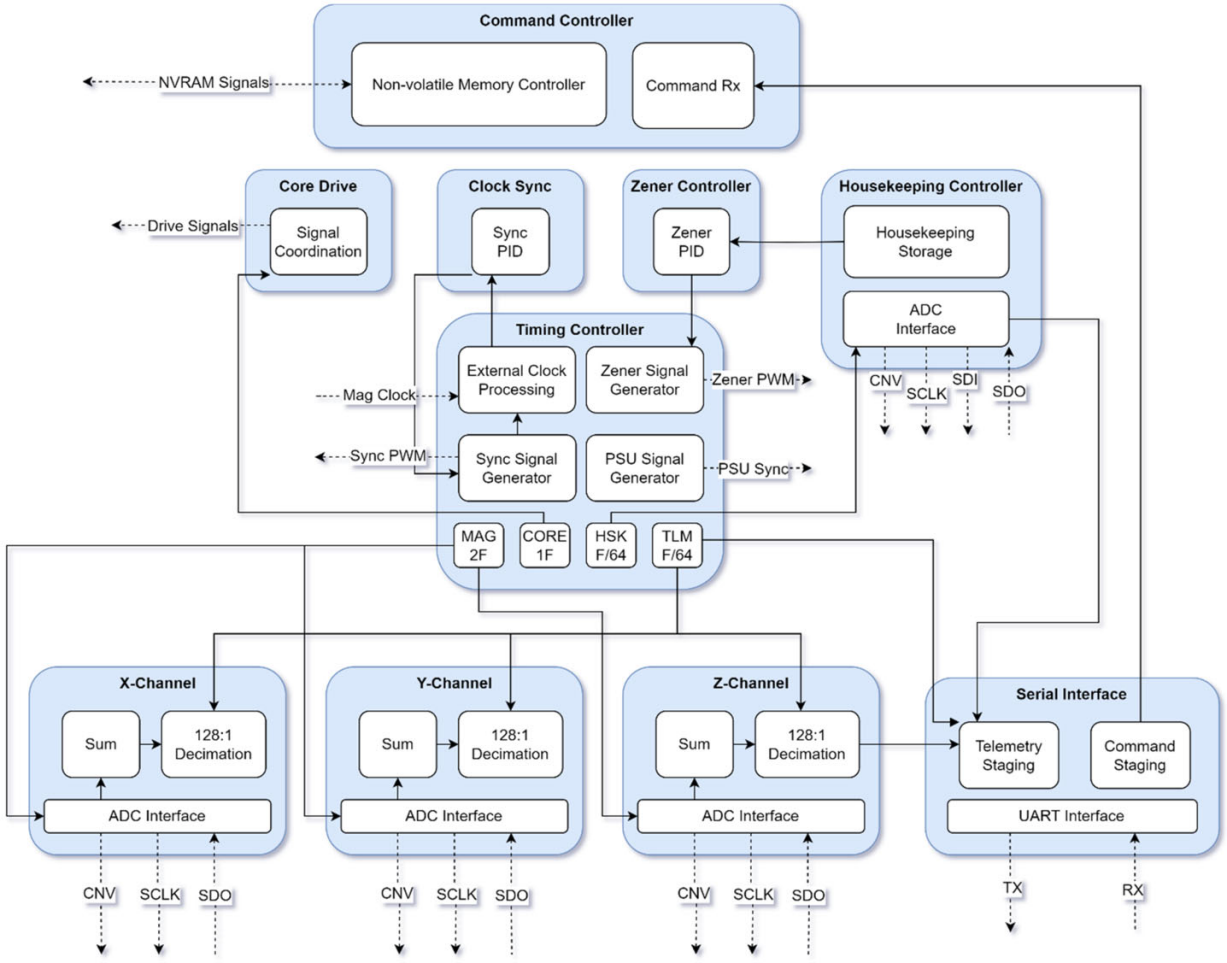
FPGA Control Logic Design Diagram

Figure 15 shows the major functions of the MAGIC FPGA control logic. The timing controller manages clock generation and coordinates all signals on a shared clock. It triggers each component at their defined frequencies, all synchronized to the fundamental 8192 Hz (1F) of the drive circuit. The FPGA drives the magnetometer cores, captures the resulting sensor output, gathers housekeeping data, synchronizes them to the MAG instrument, saves and loads state to non-volatile memory, and packetized data for telemetry. Magnetometer channels are sampled at 128 times the telemetered frequency; these samples are summed and reduced to 20-bit width for the science product.

### Electronics Box

**Fig. 16:**
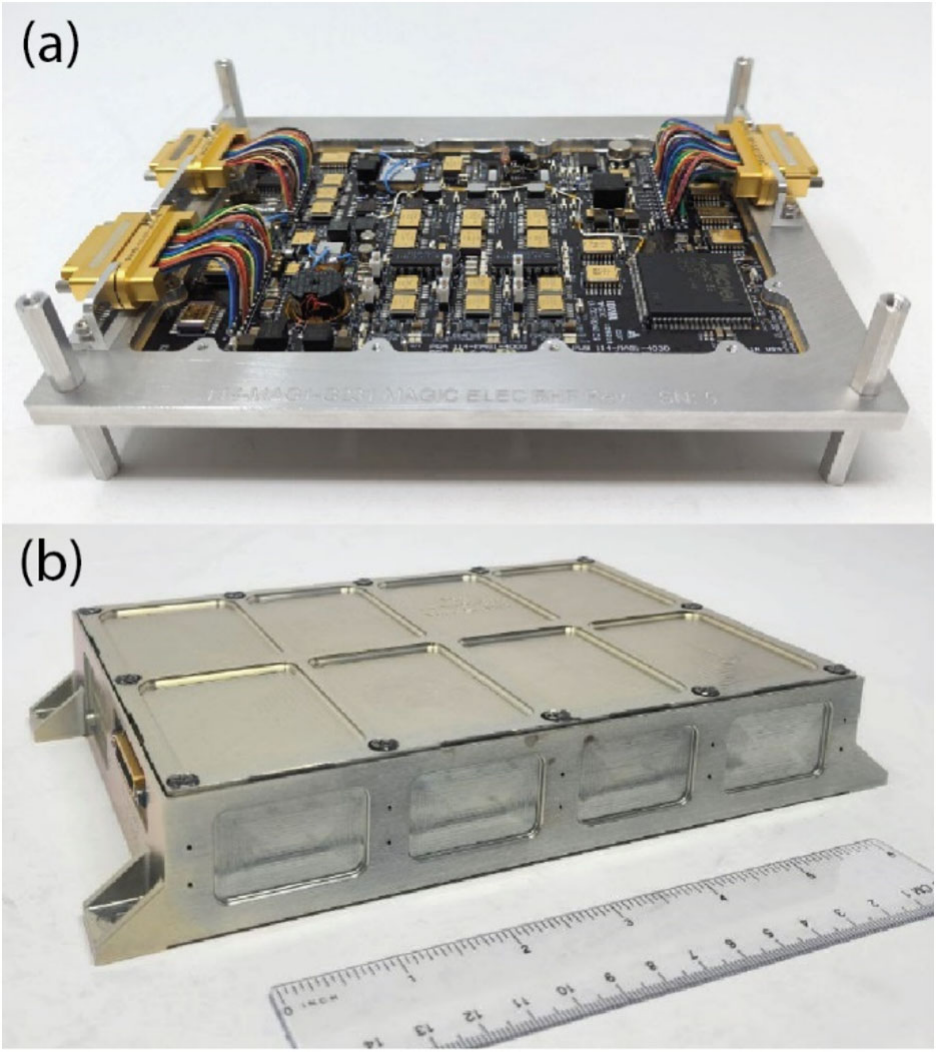
MAGIC’s electronics ($a$) are accommodated in a dedicated electronics box ($b$) comprising one aluminum frame with removable top and bottom lids

The MAGIC electronics (Fig. 16, a) are accommodated in a dedicated electronics box. The printed wiring assembly is housed in a custom aluminium tray with removable top and bottom lids (Fig. 16, b). Three externally accessible micro-D connectors provide the harness to the sensor, the power and telemetry interface to the TRACERS Central Data Processing Unit (CDPU), and a debug/JTAG interface that is capped for flight. MAGIC’s electronics board is connected to the CDPU with a 31-pin connector and the sensor with a 25-pin connector with twisted pairs and a silver/copper overbraid for ESD shielding fed through Kynar tubing.

## Design Changes and Challenges

MAGIC was originally intended to be mounted 50 cm from the spacecraft in the center of a two-segment 100 cm deployable magnetometer boom with the primary MAG sensor at the tip. However, a late emerging technical issue required to mission to pivot to the fixed magnetometer brackets shown in Fig. 20. The reduced ∼20 cm separation from the spacecraft will significantly enhance the apparent magnetic noise from the spacecraft as MAGIC is now likely in the near-field multi-pole region of several significant stray magnetic field sources including the time-varying field from the solar arrays modulated at the spin frequency of the spacecraft. Significant effort has been made to characterize both the AC and time-varying fields generated by different spacecraft subsystems (Miles et al. 2025b) to inform their removal in post-processing using modern signal processing techniques (Finley et al. 2023a,b, 2024; Hoffmann et al. 2024)

It was discovered during thermal stability testing on EM Ringcore hardware that extremely cold temperatures influenced the overall sensitivity of the sensor. This led to updates to the passive components in the transconductance amplifier to balance the thermal stability, noise floor, range and linearity of the sensor.

During the Ringcore sensor TVAC testing it was found that the instruments input current increased at high temperatures. This was unique to this sensor and electronics pair and was not repeatable on engineering versions of the hardware. This was attributed, based on available housekeeping data, to degradation of the resonant tuning of the core drive circuit resulting in increased drive power dissipation. Thermal analysis showed that the temperatures at which this occurred would not be encountered on-orbit during science conditions, so the instrument was flown as-is.

## Calibration

Fluxgate magnetometers construct their estimate of the in-situ vector magnetic field from three nominally orthogonal measured components that are subject to variations in gain, relative orientation, and offset over time and temperature. These error sources can be minimized and their variation over time and temperature reduced, but the errors cannot be fully eliminated. Calibration was used to correct the raw data from each sensor into the fully qualified data product. Each sensor’s characteristics were documented through a series of specialized tests before delivery.

MAGIC uses a vector calibration (Broadfoot et al. 2022) to transform the raw data from each sensor ($E$, in engineering units) into a robust measurement of the in-situ magnetic field in the common reference frame of the spacecraft ($B_{\mathrm{CRF}}$, in nT). The raw sensor data are assumed to have error in offset ($\boldsymbol{b}$), sensitivity ($\boldsymbol{S}$), orthogonality ($\boldsymbol{P}$), and rotation ($\boldsymbol{R}_{\boldsymbol{A}} $) that are first determined via pre-flight characterization and then trended via in-situ calibration. These 12 basic calibration parameters (3 offset, 3 sensitivity, 3 orthogonality, and 3 Euler angles) provide the calibrated magnetic field vector in the common reference frame from the sensor using $\boldsymbol{B}_{\boldsymbol{\mathrm{CRF}}} = \boldsymbol{R}_{\boldsymbol{A}}^{-1} \boldsymbol{P}^{-1} \boldsymbol{S}^{-1} \left ( \boldsymbol{E} - \boldsymbol{b} \right )$.

Obtaining these directly involves solving a set of non-linear equations that are dependent on initial parameters. However, following Olsen et al. (2020) this can be rewritten as $\boldsymbol{R}_{\boldsymbol{A}}^{-1} \boldsymbol{P}^{-1} \boldsymbol{S}^{-1} \left ( \boldsymbol{E} - \boldsymbol{b} \right ) = \boldsymbol{AE}+ \tilde{\boldsymbol{b}}$ where $\boldsymbol{A}=\ \boldsymbol{R}_{\boldsymbol{A}}^{-1} \boldsymbol{P}^{-1} \boldsymbol{S}^{-1}$ is a 3x3 matrix and $\tilde{\boldsymbol{b}} =\ - \boldsymbol{Ab}$. This allows the equation to be solved as a linear inverse problem which is no longer dependent on initial assumptions. The calibration parameters are then extracted by reforming the linearized results of $\boldsymbol{A}$ into matrix form. Matrix decomposition of the resultant calibration matrix to an orthonormal (Q) and lower triangular matrix (L), knows as QL decomposition (Houtzager 2021), allows for the underlying instrument parameters (sensitivity, orthogonality, rotation, and offset) to be trended for analysis and verification.

Three other instrument effects are corrected inside of this vector calibration: the minor non-linearity of each channel, the weak dependency of gain on sensor temperature, and the weak dependency of instrument zeros on electronics temperature. Linearity is characterized in pre-flight calibration and then periodically using the in-situ calibration mode. A polynomial fit to this trend affects a minor linearization of the raw engineering unit data before vector calibration. The minor (∼5 ppm) dependence of the sensitivity on the sensor temperature is established via pre-flight calibration and then linearly corrected. The weak dependences of the zero-offset on the electronics temperature are similarly established via pre-flight calibration and linearly corrected.

**Fig. 17:**
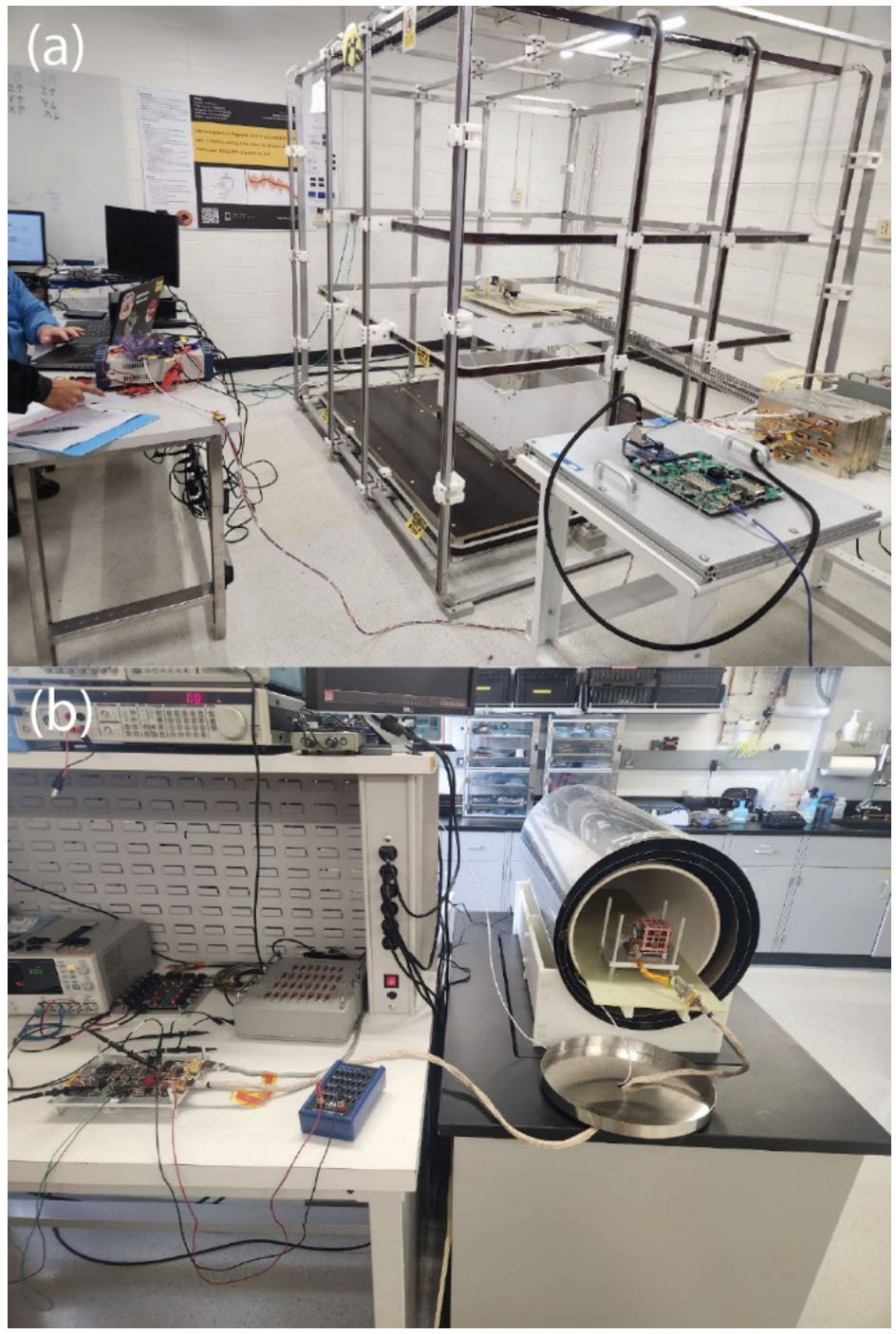
($a$) Preflight calibration was performed in 2 m actively compensated three-axis Merritt coil at the University of Iowa. ($b$) Zero-offset estimation was performed using sensor-flips in near-zero field inside a multi-layer magnetic shield

Pre-launch calibrations were completed to measure the instrumental zeros and the orthogonality and sensitivity matrices for both MAGIC instruments using the three-axis actively compensated Merritt coil facility at the University of Iowa (Fig. 17, a). This apparatus can apply a controlled vector magnetic field in a meter cubic test volume. Large-amplitude staircase test signals in each component further allow an independent estimate of the linearity of each sensor axis. Finally, the zero-offsets were established using the sums-and-differences technique where the sensor was placed in the near-zero field inside a multilayer mumetal magnetic shield (Fig. 17, b) and flipped in each axis (analogous to using the spacecraft’s on-orbit spin to calibrate the two instrument components orthogonal to the spin axis).

Prior to vector-vector or scalar-vector calibrations, an expected line of best fit is calculated for the large amplitude staircases. The residuals of the expected linear response from the applied staircase test pattern determines the non-linear characteristics of each axis. A polynomial fit of third or fifth order corrects this nonlinear response. Table 2 shows the linearity coefficients for each axis of each instrument fit to the equation $\boldsymbol{E}_{\boldsymbol{i},\boldsymbol{\mathrm{corrected}}} = \boldsymbol{\Sigma c}_{\boldsymbol{n}}\boldsymbol{E}_{\boldsymbol{i}}^{\boldsymbol{n}}$.

**Table 2:** Linearity coefficients for each sensor axis. A lowest order of 3 was utilized where applicable

	*Tesseract linearity coefficients*			*Ringcore linearity coefficients*		
	*X*	*Y*	*Z*	*X*	*Y*	*Z*
*c*_*5*_	0	1.7167E-29	0	*1.0683E-29*	*3.1051E-30*	*4.5126E-30*
*c*_*4*_	0	2.0937E-24	0	*-1.5143E-24*	*-5.4491E-24*	*-9.085E-24*
*c*_*3*_	2.6311E-16	-8.7347E-16	2.4617E-16	*-2.5540E-16*	*2.7585E-17*	*-1.9997E-17*
*c*_*2*_	1.2565E-10	3.6193E-11	5.9773E-11	*-5.4107E-13*	*1.6956E-10*	*2.5138E-10*
*c*_*1*_	0.9949	1.0089	0.9953	*1.00128*	*0.9990*	*0.9994*

Calibration as performed after the linearity correction to determine the sensitivity, orthogonality, and offsets (Table 3). Both scalar-vector and vector-vector calibrations performed in the University of Iowa Merritt coil provide offsets which are a sum of the sensor and the surrounding lab space. To determine the physical sensor offsets, sensors are placed in a mumetal shield can and physically flipped 180^∘^. The average of the resulting measurements cancels the near-zero field of the magnetic shield and provides the sensor offset using the prior calibration matrix $\boldsymbol{A}$. Calculating the field within the mumetal shielding can shows a consistent value between the sensor flips near zero and thus confirms a good calibration of the sensor offsets.

**Table 3:** Sensitivity, orthogonality, and offset coefficients determined by a vector-vector and scalar-vector calibration. Euler angles are provided by vector-vector calibration; however, scalar-vector calibrations are a rotation-less calibration and therefore do not have Euler angles. Offsets calculated generated using sensor flips in a magnetic shield

	Tesseract	Ring core
S_x_	0.96266	1.29751
S_y_	1.02989	1.15127
Sz	0.94234	1.19307
*φ*	0.25100	0.36446
*ρ*	-0.01084	-0.15397
*λ*	-0.06468	0.02988
O_X_	93.9	192.6
O_Y_	-503.3	-223.9
O_Z_	178.2	-372.7
E_1_	-0.30967	-0.45739
E_2_	-0.11045	-0.91182
E_3_	2.00783	-0.77714

**Fig. 18:**
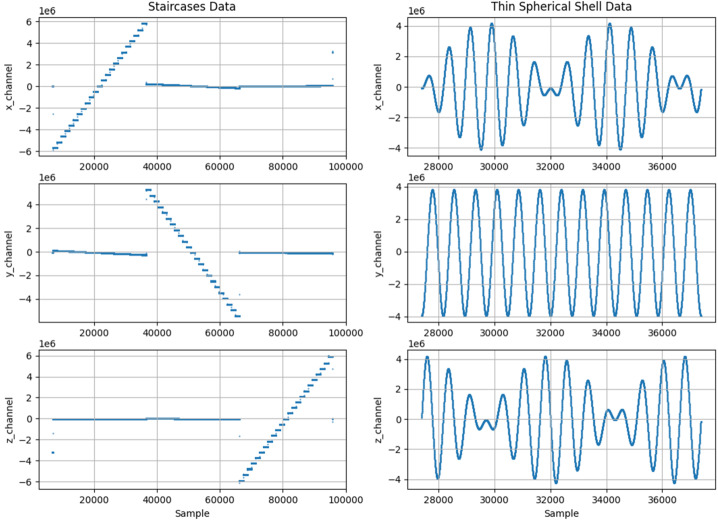
Example calibration data as recorded by the Tesseract instrument sensor and displayed in E. The left column depicts a series of staircases ranging from ±55,000 nT used for the VV calibration while the right column depicts a thin spherical shell of 40,000 nT magnitude used for the SV calibration

Due to the stochastic nature of the calibration, both vector-vector and scalar-vector calibrations are computed with the intent of determining a physical fit of the coefficients, where both calibrations should collapse to the same coefficients. Scalar-vector calibrations fit a linear parameterized model $\boldsymbol{B}^{2} -\boldsymbol{B}_{0}^{2} = (\boldsymbol{E}\cdot \boldsymbol{E}) \cdot \boldsymbol{p}$, where the vector $\boldsymbol{p} $ represents a solution which converts $\boldsymbol{E}$ into the reference magnetic field (Merayo et al. 2000). However, $\boldsymbol{B}$ may also be expressed, as with a vector-vector calibration described earlier, as a matrix multiplication $\boldsymbol{B} = \boldsymbol{A}[\boldsymbol{E} - \boldsymbol{b}]$, allowing matrix $\boldsymbol{A}$ to be solved from vector $\boldsymbol{p}$. Figure 18 shows the type of data utilized for the calibrations, where the staircases provided a vector-vector calibration and the thin shell profile provided a scalar-vector calibration.

**Fig. 19:**
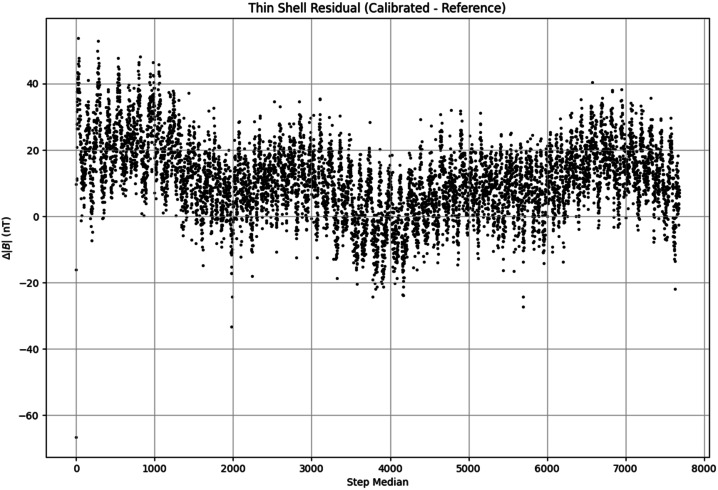
Example residuals from the scalar-vector calibration on Tesseract

Figure 19 show the residuals of a thin-shell scalar vector calibration. In an idealized environment and a perfect sensor, the residuals should be zero, however sensor noise and background noise in the lab environment introduce errors which show as non-zero residuals.

On-orbit, MAGIC will trend its calibrations via in-situ vector-vector calibration against the CHAOS magnetic field model (Finlay et al. 2020) during geomagnetically quiet conditions. Proximity of MAGIC to the spacecraft introduces a non-negligible magnetic noise within the measurements provided by the instrument. A magnetic screening of the spacecraft will determine the expected offset at MAGIC location and interference mitigation techniques will be applied in the level 3 data product to account for these offsets.

## MAGIC Operation

MAGIC has one normal operation mode; the instruments continuously measure the vector magnetic field when powered and stream magnetic field measurements with housekeeping data sub-commutated at low cadence. Magnetic data is telemetered at full 128 sps cadence to the Main Electronics Box (MEB). Science data is telemetered to the ground at full cadence during the Region of Interest (ROI). It is reduced by onboard software processing to 16 sps during the back-orbit. Instrument function can be changed by the upload of table parameters which can be saved into non-volatile memory. However, no commanding requirements are required for normal operation.

### Spacecraft Accommodation

**Fig. 20:**
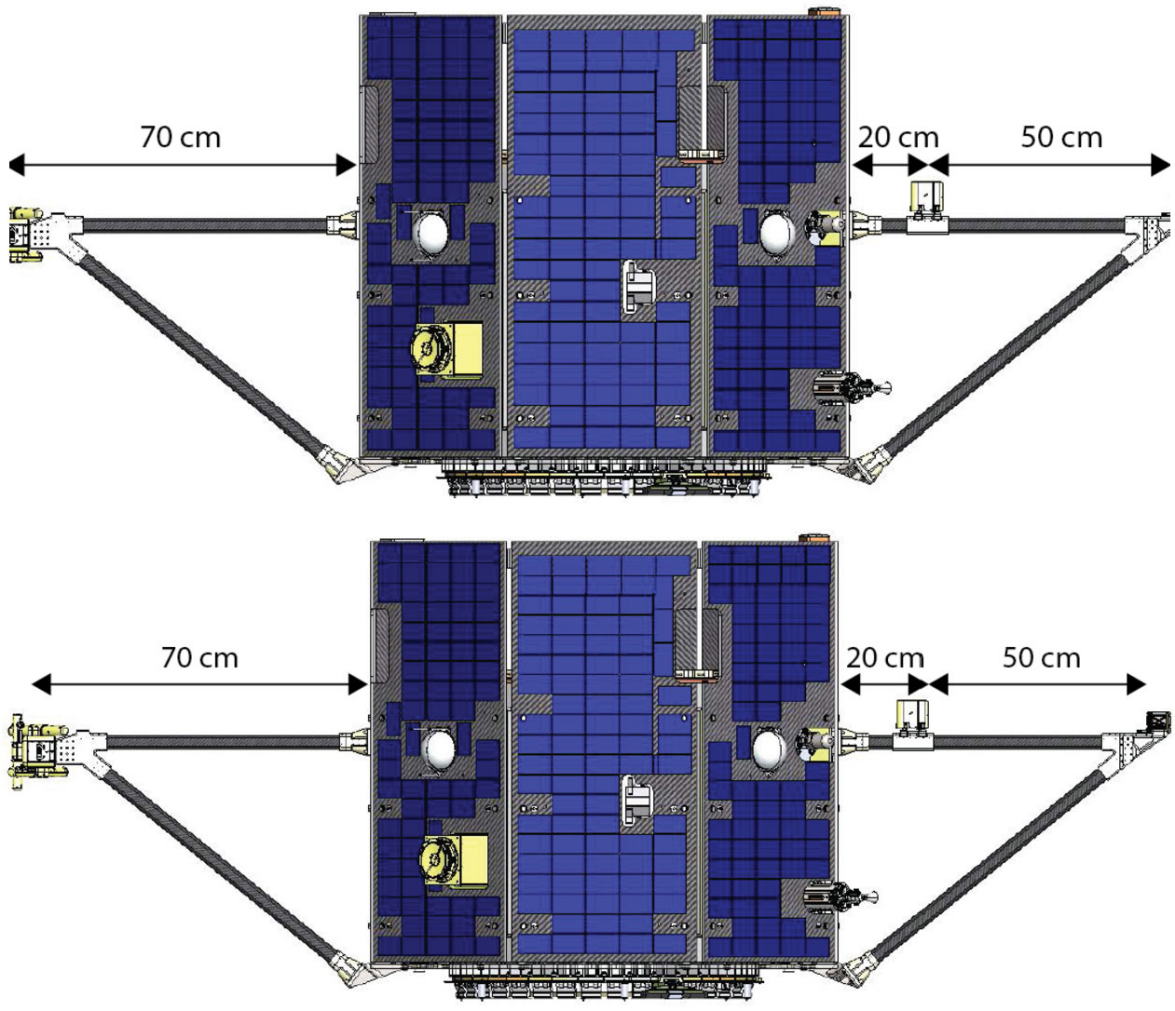
TRACERS spacecraft showing the accommodation of the MAG at 70 cm from the spacecraft face and MAGIC at 20 cm. MSC provides spin-balance on an opposing bracket with the single heavier sensor at 70 cm. Spacecraft image credit Millenium Space Systems

MAGIC’s integration into the pre-existing TRACERS spacecraft was straightforward and required minimal changes. The MAGIC sensors were accommodated on the same magnetometer bracket as the primary MAG sensor (Fig. 20). MAGIC is mounted about 20 cm from the nearest spacecraft solar panel, far enough to attenuate the worst of the strong near-field magnetic interference, and 50 cm from the MAG sensor to minimize the inter-instrument interference. The MSC search coil magnetometer is bracket deployed opposite the MAG sensor. The MSC induction coil sensor is heavier that the MAG sensor, so each TRACERS spacecraft originally budgeted ∼700 g of ballast for spin-balance. The MAGIC sensor, mounted inboard of MAG, replaced a portion of this ballast.

The most significant impact of MAGIC’s accommodation is the additional requirement to characterize the presence of two fluxgate sensors on the calibration coefficients of each. Both fluxgate designs use magnetic feedback in the sensor to linearize and stabilize their magnetic sensitivity. Consequently, when one fluxgate is powered on it creates small, but not trivial, gain-error in the other instrument due to the stray magnetic-feedback field from the sensor. This is a known effect of multi-sensor fluxgate arrays and can be resolved by characterizing the gain of each sensor individually and then of both sensors in their flight configuration. A series of cross-calibration activities were used to quantify the small effect that MAGIC’s presence has on the MAG’s gain and confirm that it can be corrected via calibration (Miles et al. 2025b). The cross-calibration activities identified a linear relation between the observed external field and the interference observed by MAG from MAGIC of ∼5nT in a 20,000 nT external field.

### Electronics Box Accommodation

**Fig. 21:**
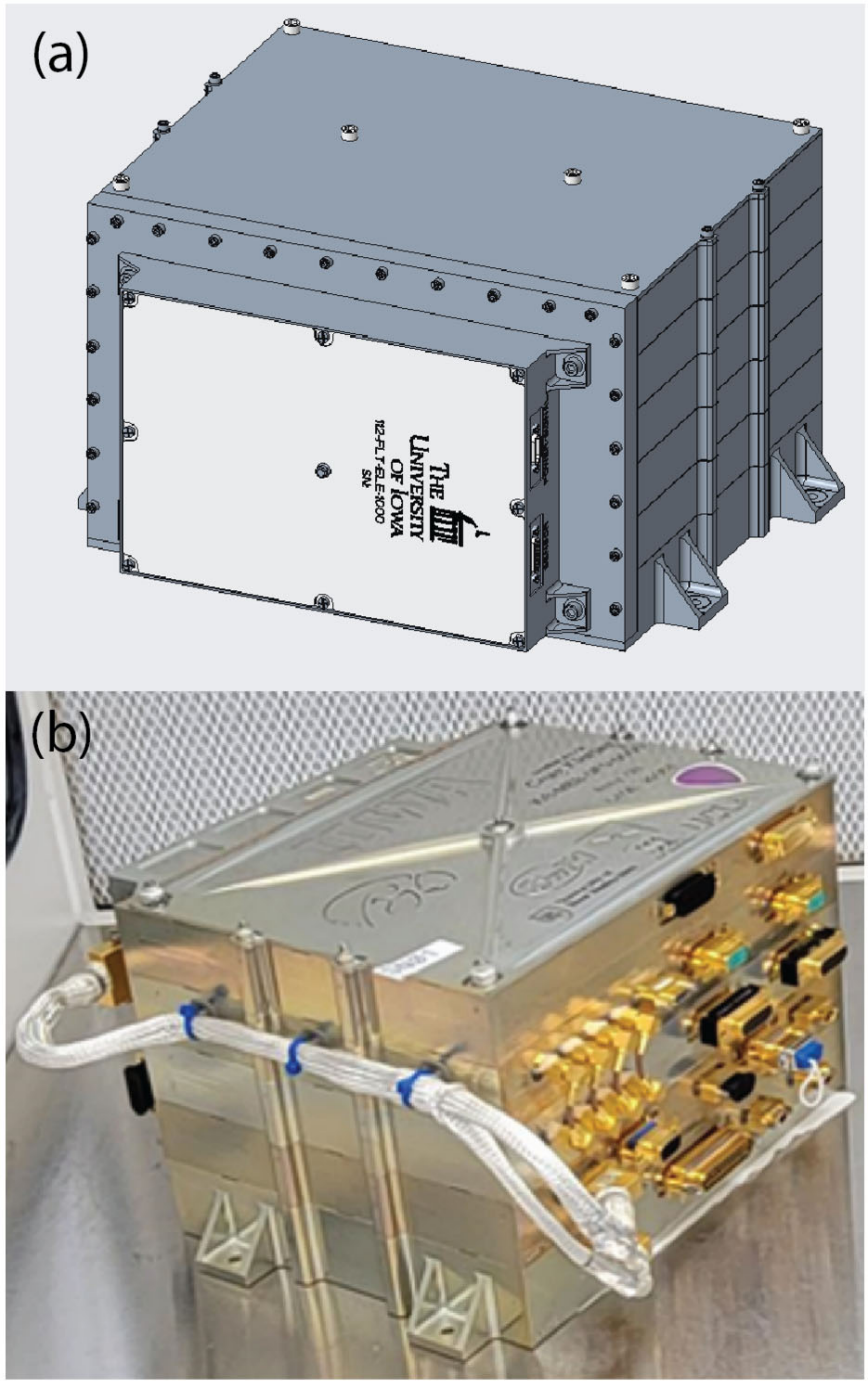
The MAGIC electronics stand-alone e-box mounted to the side of the main electronics box

The MAGIC electronics are housed in small electronics box (Fig. 21) mounted to the side of the TRACERS Instrument Suite (TIS) Main Electronics Box (MEB). MAGIC presents three connectors: a sensor cable, a power and data cable, and a debug/development connector that is capped for flight.

### Electrical Accommodation

**Fig. 22:**
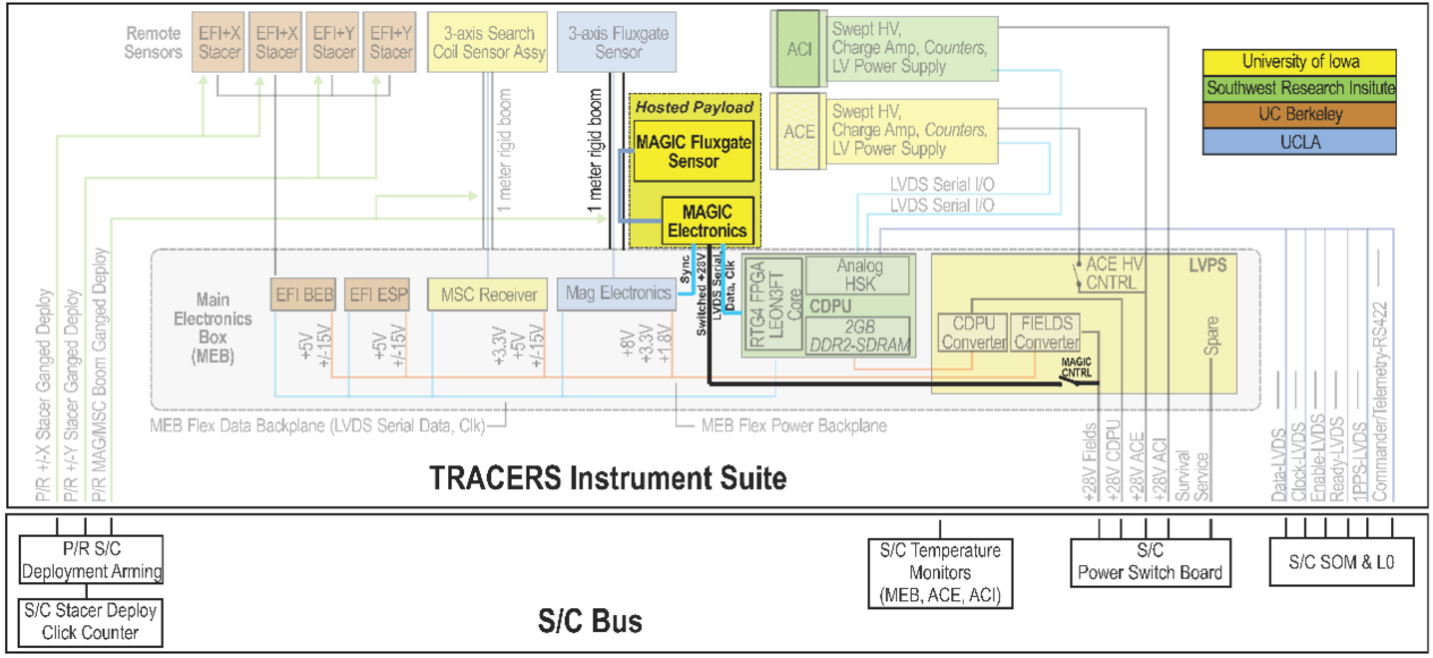
MAGIC integrates to the TRACERS main electronics box via power and telemetry connectors and is powered by a switched electrical service that can be shut off if MAGIC impacts the TRACERS mission

Figure 22 shows the electrical interface to the MAGIC payload. MAGIC incorporates a synchronous isolated 28 V power converter that produces all required internal regulated voltages. Onboard regulation also allows MAGIC to be easily accommodated on a do-no-harm basis. MAGIC is powered by a dedicated 28 V feed switched by the MEB. This allows MAGIC to be completely de-energized should its operation be found to impact the primary mission on-orbit.

MAGIC provides a low-speed serial data interface to transmit science and housekeeping data to the MEB and receive any required commands via LVDS. MAGIC requires a standard 1 pulse-per-second (PPS) signal for high-precision timing. MAGIC additionally requires a drive synchronization signal, produced by the primary MAG payload. If this signal is present, MAGIC synchronizes the excitation signal used to drive the MAGIC sensor cores through magnetic saturation. This synchronization prevents the two fluxgate instruments from mutually interfering due to their drive and sampling beating past each other. If the signal is absent, then MAGIC free runs based on an internal oscillator.

### Telemetry

MAGIC modestly increases the overall TRACERS telemetry budget. MAGIC is telemetered as 128 sps vector data (5.89 kbps) during the region of interest (magnetospheric cusp) and 16 sps vector data (0.904 kbps) during the back orbit to aid instrument characterization and calibration. Instrument housekeeping creates an additional 0.56 kbps. Data from the MAGIC payload is sent by a low-speed serial link to the Main Electronics Box (MEB) for processing and decimation. MAGIC uses the TRACERS architecture where the MEB/CDPU performs all instrument data acquisition and Consultative Committee for Space Data Systems (CCSDS) format packetization with 128 samples per packet. If the MAGIC instrument is not operating, the overall TRACERS data handling architecture inserts fill data to preserve the standard data format. This makes the spacecraft indifferent to the presence or absence of the MAGIC data, as the spacecraft bus is a bent pipe from the MEB during downlink.

### Commanding

MAGIC operates continuously in a single mode unless commanded by the CDPU. During nominal operation (science main mode), MAGIC has two modes per orbit: one utilized in the Region of Interest (ROI) and one in the remainder of the orbit. MAGIC’s operation is the same, continuously producing 128 sps data, the difference is solely in how the data is reduced in the MEB. Full cadence data are telemetered for cusp encounter regions of interest. The CDPU decimates MAGIC’s telemetry to return a low rate of data for trending and calibration purposes from the remainder of the orbit.

## Data Products

MAGIC’s science data products are vector magnetic field measurements that are built up, level by level, starting with receipt of Level 0 MAGIC CCSDS packets at the TRACERS Science Operations Center (SOC).

Level 0 comprises the raw CCSDS packets from the instrument containing science data with housekeeping interleaved at lower cadence.

Level 1a comprises the unpacked CCSDS packets with each measurement timed based on the real time spacecraft clock and the offset to the pulse-per-second timing signal.

Level 1b incorporates the provisional attitude, provisional ephemeris, the spin pulse, and static calibrations. Science data are rotated to account for physical magnetometers offsets and are provided in both the satellite, de-spun satellite frame, and geophysical (GSM, GSE, etc.) frames. Level 1b data utilize the best-known static calibration values to convert to pseudo-nT units in satellite, de-spun satellite, and geophysical coordinates and to generate quick look plots.

Level 2 data are calibrated in-situ against the CHAOS-7 magnetic field model during geophysical quiet times. Calibrated data are released in satellite and geophysical coordinates and may be suitable for distribution to the wider scientific audience for analysis and publication but may be improved by further processing to remove local magnetic noise from the spacecraft or other instruments.

Level 3 data are further processed with a combination of statistical signal processing and machine learning to remove platform magnetic noise. Level 3 data are provided in satellite, de-spun satellite, and geophysical coordinates and are suitable for research and publication.

## Dual-Sensor Gradiometry and Magnetic Noise Removal

**Fig. 23:**
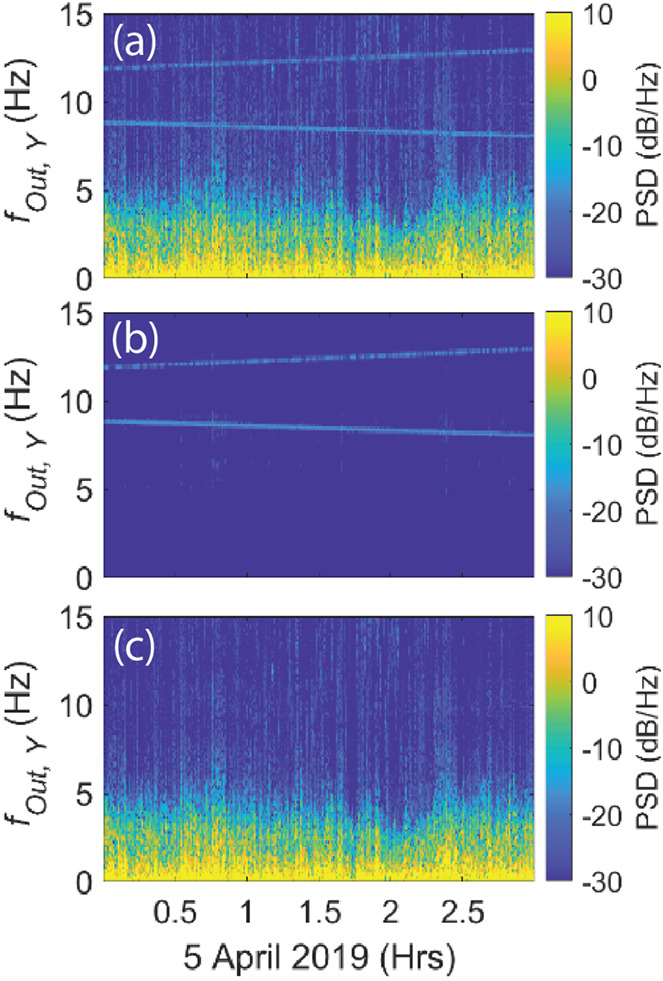
($a$) Raw Parker Solar Probe data, ($b$) extracted noise from the time-varying reaction wheels used to control spacecraft attitude, and ($c$) corrected science data with the noise removed

Mounting the existing MAG and MAGIC sensors at different distances from the spacecraft on a common bracket enables differential measurements of the local field providing an independent method of estimating the quasi-DC and dynamic spacecraft magnetic noise sources. MAGIC uses the MAGSTAR software suite (Finley et al. 2023a,b), developed in-part for TRACERS, to mitigate the residual noise at the MAGIC sensor location. MAGSTAR combines statistical signal processing and machine learning driven by the gradient between the MAG and MAGIC sensors to mitigate local interference by an expected ∼90% (e-POP and Parker Solar Probe, Fig. 23). MAGSTAR does not rely on physics-based models or housekeeping data. The combination of a bracket accommodation, gradiometer, and signal process provides robust magnetic data without imposing costly magnetic controls on the other instruments or the commercial nanosatellite.

## Benefits and Lesson’s Learned from a Technology Demonstration Flight

Like all new space instruments, MAGIC needed flight heritage to be credibly proposed for most future NASA applications. Sub-orbital sounding rockets or nanosatellite deployments provide some heritage. However, the gold standard is on-orbit production of robust and scientifically useful long-term data on a high-reliability spacecraft mission.

**Fig. 24:**
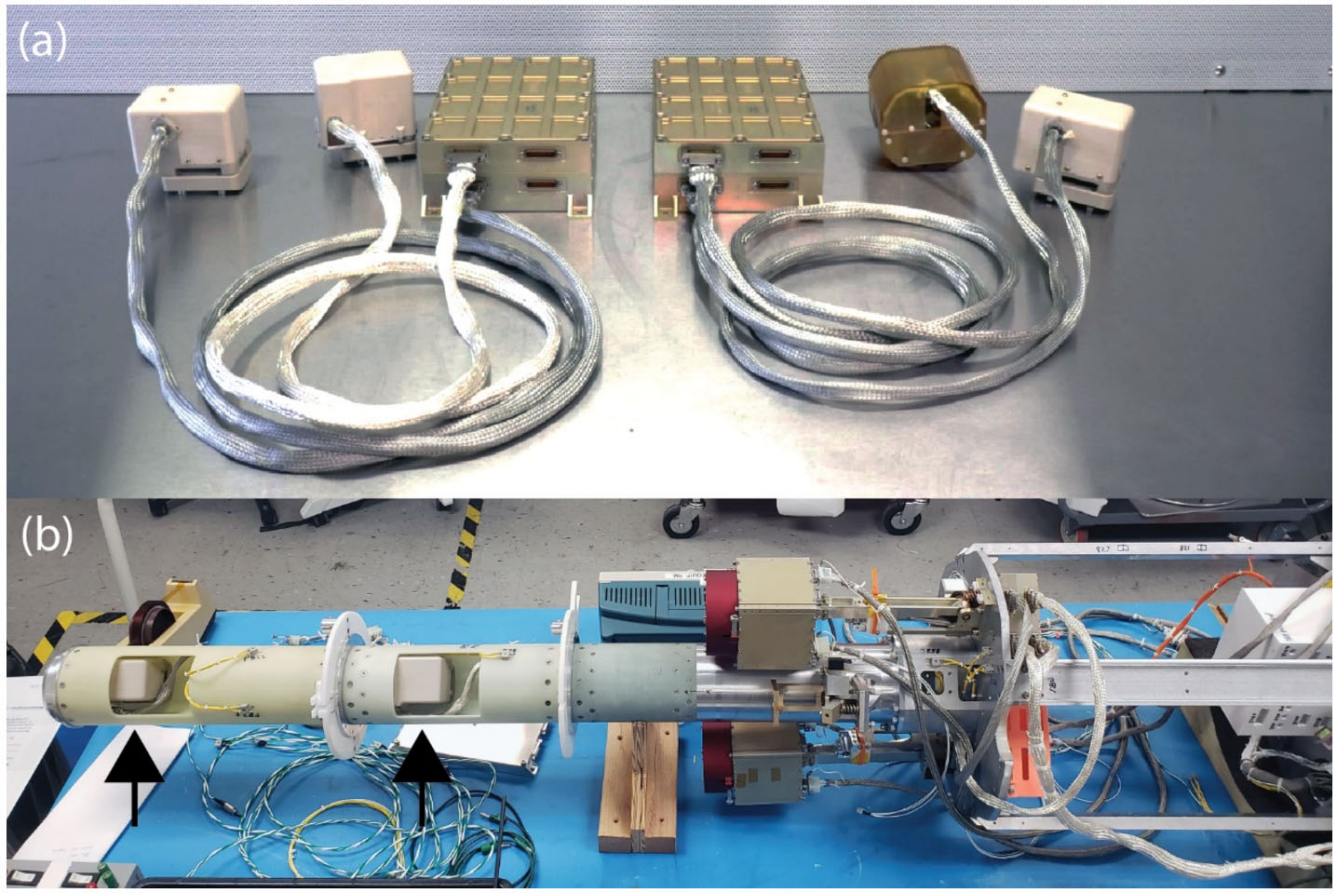
Four magnetometers ($a$) for the two 2022 ACES-II rockets ($b$) launched November 2022

The first demonstration flight for the MAGIC design was four instruments (Fig. 24) for ACES-II. They were successfully integrated at NASA Wallops in August 2022, passed sounding rocket environmental qualification, and all four instruments operated successfully in flight in December 2022 on the two sounding rockets (Greene et al. 2024, 2025).

The MAGIC team comprises primarily early career scientists and engineers providing an exceptional opportunity to develop personnel for future missions. MAGIC is staffed similarly to a standard NPR 7120.5D instrument team but tailored and streamlined for the reduced requirements of a technology demonstration.

The MAGIC PI has full responsibility for the leadership, management, scientific integrity, implementation, development, and science return for the MAGIC instrument through all phases. The PI reports and to the Explorers Program Office for the portion of this work related to the flight on the TRACERS SMEX mission. The PI is assisted by a postdoc to develop future expertise in experimental space physics and magnetometer instrumentation. An instrumentation focused PhD student participates in the design, analysis, construction, and calibration.

**Fig. 25:**
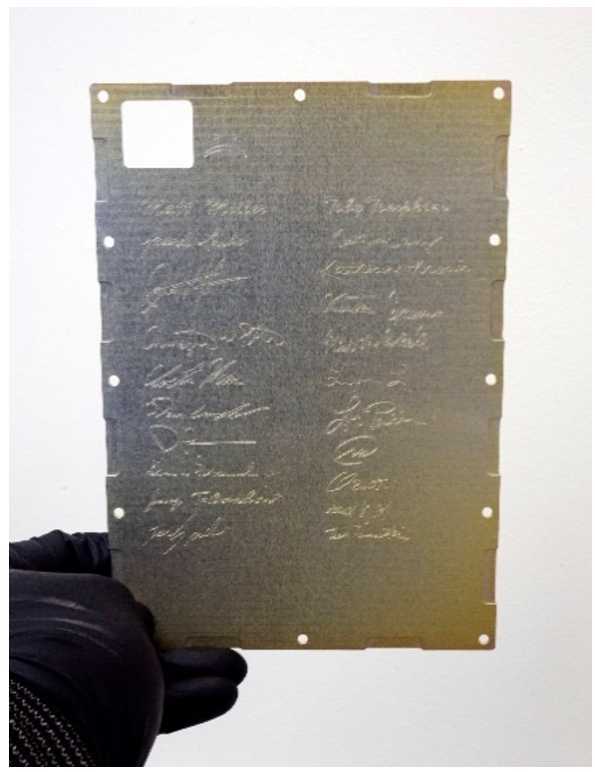
MAGIC is the first piece of flight hardware delivered by most of the early-career MAGIC team and their signatures are laser etched to the lid of each electronics box

MAGIC was a new instrument in the University of Iowa’s spaceflight instrumentation portfolio and required additional technical and scientific staff. The MAGIC team is a diverse and early career group of scientists and engineers designed to enable long-term capability to design, build, and test next-generation spaceflight fluxgates. For most of the team, this was their first spaceflight hardware project (Fig. 25). Early-career staff were guided by appropriate senior mentors.

The MAGIC project management (PM) and systems engineering (SE) roles are combined into a single staff member. MAGIC used a dedicated electrical engineer, mechanical engineer, firmware engineer, and a fractional effort of a technician. All early-career staff had an identified mentor from the existing senior engineering staff in the Department of Physics and Astronomy. Other roles (configuration control, quality assurance, assembly, software, parts, etc.) were provided by matrix-managed part-time effort from existing staff.

## Conclusions

MAGIC will flight demonstrate new fluxgate magnetometer core technology that provides an alternative to the legacy cores that have been routinely used for spaceflight instruments. The two TRACERS spacecraft carry different sensor variants – one using traditional 1” ring-cores compatible with the S1000 standard and the other using a new racetrack design. MAGIC will produce fully calibrated science data products and, in concert with the primary MAG instrument, provides the potential for gradiometer driven signal processing to help mitigate stray magnetic fields from the spacecraft and other instruments. MAGIC was designed, built, and tested by a team of early career scientists and engineers showing how technology demonstrations are an exceptional platform to train next-generation space professionals in addition to demonstrating next-generation technology.

## References

[CR1] Acuña MH, Scearce CS, Seek J, Scheifele J (1978) The MAGSAT vector magnetometer: a precision fluxgate magnetometer for the measurement of the geomagnetic field. Technical Memorandum 79656. National Aeronautics and Space Administration. https://ntrs.nasa.gov/citations/19790010349

[CR2] Broadfoot RM, Miles DM, Holley W, Howarth AD (2022) In situ calibration of the Swarm-Echo magnetometers. Geosci Instrum Method Data Syst 11:323–333. 10.5194/gi-11-323-2022

[CR3] Finlay CC, Kloss C, Olsen N, Hammer MD, Tøffner-Clausen L, Grayver A, Kuvshinov A (2020) The CHAOS-7 geomagnetic field model and observed changes in the South Atlantic Anomaly. Earth Planets Space 72:156. 10.1186/s40623-020-01252-9 33122959 10.1186/s40623-020-01252-9 PMC7578192

[CR4] Finley MG, Bowen TA, Pulupa M, Koval A, Miles DM (2023b) Statistical decomposition and machine learning to clean in situ spaceflight magnetic field measurements. Geophys Res Lett 50:e2023GL103626. 10.1029/2023GL103626

[CR5] Finley MG, Broadfoot RM, Shekhar S, Miles DM (2023a) Identification and removal of reaction wheel interference from in-situ magnetic field data using multichannel singular spectrum analysis. J Geophys Res Space Phys 128:e2022JA031020. 10.1029/2022JA031020

[CR6] Finley MG, Flores AM, Morris KJ, Broadfoot RM, Hisel S, Homann J, Piker C, Sen Gupta A, Miles DM (2024) Enabling in situ validation of mitigation algorithms for magnetic interference via a laboratory-generated dataset. Geosci Instrum Method Data Syst 13:263–275. 10.5194/gi-13-263-2024

[CR7] Forslund Å, Belyayev S, Ivchenko N, Olsson G, Edberg T, Marusenkov A (2007) Miniaturized digital fluxgate magnetometer for small spacecraft applications. Meas Sci Technol 19:015202

[CR8] Greene K, Hansen C, Narod BB, Dvorsky R, Miles DM (2022) Tesseract – a high-stability, low-noise fluxgate sensor designed for constellation applications. Geosci Instrum Method Data Syst 11:307–321. 10.5194/gi-11-307-2022

[CR9] Greene K, Bounds SR, Broadfoot RM, Feltman C, Hisel SJ, Kraus RM, Lasko A, Washington A, Miles DM (2024) First in situ measurements of the prototype Tesseract fluxgate magnetometer on the ACES-II-Low sounding rocket. Geosci Instrum Method Data Syst 13:249–262

[CR10] Greene K, Miles DM, Bounds SR, Bonnell JW, Feltman C, Roglans R, Streltsov A (2025) In situ evidence of ionospheric feedback instability adjacent to a quiescent auroral arc. Geophys Res Lett 52:e2024GL110479. 10.1029/2024GL110479

[CR11] Hoffmann AP, Moldwin MB, Imajo S, Finley MG, Sheinker A (2024) MAGPRIME: an open-source library for benchmarking and developing interference removal algorithms for spaceborne magnetometers. Earth Space Sci 11:e2024EA003675. 10.1029/2024EA003675

[CR12] Houtzager I (2021) QR/RQ/QL/LQ factorizations. GitHub. https://github.com/iwoodsawyer/factor

[CR13] Merayo JMG, Brauer P, Primdahl F, Raagaard Petersen J, Nielsen OV (2000) Scalar calibration of vector magnetometers. Meas Sci Technol 11(2):120.

[CR14] Miles DM, Mann IR, Kale A, Milling DK, Narod BB, Bennest JR, Barona D, Unsworth MJ (2017) The effect of winding and core support material on the thermal gain dependence of a fluxgate magnetometer sensor. Geosci Instrum Method Data Syst 6:377–396

[CR15] Miles DM, Ciurzynski M, Barona D, Narod BB, Bennest JR, Kale A, Lessard M, Milling DK, Larson J, Mann IR (2019) Low-Noise Permalloy Ring-Cores for Fluxgate Magnetometers. Geosci Instrum Method Data Syst: 1–20. 10.5194/gi-2019-15

[CR16] Miles DM, Dvorsky R, Greene K, Hansen CT, Narod BB, Webb MD (2022) Contributors to fluxgate magnetic noise in permalloy foils including a potential new copper alloy regime. Geosci Instrum Method Data Syst 11:111–126. 10.5194/gi-11-111-2022

[CR17] Miles DM, Kletzing CA, Fuselier SA, Goodrich KA, Bonnell JW, Bounds S, Cao H, Chen LJ, Christopher IW, Cleveland K, Connor HK, Crawford D, Dolan JS, Dorelli JC, Dvorsky R, Finley MG, Øieroset M, Petrinec SM, Phillips ML, Powers B, Prasasd R, Rospos A, Santolik O, Strangeway RJ, Trattner KJ, Washington A (2025a) The tandem reconnection and cusp electrodynamics reconnaissance satellites (TRACERS) mission. Space Sci Rev 221:61. 10.1007/s11214-025-01184-4 40584404 10.1007/s11214-025-01184-4 PMC12204947

[CR18] Miles DM, Lasko A, Blandin M, Bounds SR, Caron R, Carton A, Dolan JS, Dvorsky R, Finley MG, Flores AM, Goss C, Halekas J, Hospodarsky GB, Mark D, Marotto A, Miller I, Nguyen P, Omar S, Orrill E, Prasad R, Rospos A, Slagle A, Strangeway RJ, Vernot R, Washington A (2025b) The TRACERS Magnetic Control Plan. Space Sci Rev 221 10.1007/s11214-026-01326-2 PMC13550070 42712465

[CR19] Narod BB (2014) The origin of noise and magnetic hysteresis in crystalline permalloy ring-core fluxgate sensors. Geosci Instrum Method Data Syst 3:201

[CR20] Narod BB, Bennest JR (1990) Ring-core fluxgate magnetometers for use as observatory variometers. Phys Earth Planet Inter 59:23–28

[CR21] Narod BB, Miles DM (2024) Copper permalloys for fluxgate magnetometer sensors. Geosci Instrum Method Data Syst 13:131–161. 10.5194/gi-13-131-2024

[CR22] Olsen N., Albini G., Bouffard J., et al. (2020) Magnetic observations from CryoSat-2: calibration and processing of satellite platform magnetometer data. Earth Planets Space 72:48. 10.1186/s40623-020-01171-9

[CR23] Primdahl F (1979) The fluxgate magnetometer. J Phys E 12:241

[CR24] Primdahl F, Jensen PA (1982) Compact spherical coil for fluxgate magnetometer vector feedback. J Phys E 15:221

[CR25] Primdahl F, Nielsen OV, Petersen JR, Ripka P (1994) High frequency fluxgate sensor noise. Electron Lett 30:481–482

[CR26] Scarzello JF, Holmes JJ, O’Keefe EC (2001) Integrating fluxgate magnetometer. US Department of Navy, United States patent US 6,278,272 B1

[CR27] Snare RC (1998) A history of vector magnetometry in space. In: Pfaff RF, Borovsky JE, Young DT (eds) Measurement techniques in space plasmas fields. American Geophysical Union, pp 101–114. 10.1002/9781118664391.ch12

[CR28] Wallis DD, Miles DM, Narod BB, Bennest JR, Murphy KR, Mann IR, Yau AW (2015) The CASSIOPE/e-POP magnetic field instrument (MGF). Space Sci Rev 189:27–39. 10.1007/s11214-014-0105-z

